# Antiplasmodial dihetarylthioethers target the coenzyme A synthesis pathway in *Plasmodium falciparum* erythrocytic stages

**DOI:** 10.1186/s12936-017-1839-3

**Published:** 2017-05-15

**Authors:** Thomas Weidner, Leonardo Lucantoni, Abed Nasereddin, Lutz Preu, Peter G. Jones, Ron Dzikowski, Vicky M. Avery, Conrad Kunick

**Affiliations:** 1 Institut für Medizinische und Pharmazeutische Chemie, Technische Universität Braunschweig, Beethovenstraße 55, 38106 Braunschweig, Germany; 2 Discovery Biology, Griffith Institute for Drug Discovery, Griffith University, Nathan, 4111 QLD Australia; 3 Department of Microbiology and Molecular Genetics, IMRIC, The Kuvin Center for the Study of Infectious and Tropical Diseases, The Hebrew University-Hadassah Medical School, 91120 Jerusalem, Israel; 4 Institut für Anorganische und Analytische Chemie, Technische Universität Braunschweig, Hagenring 30, 38106 Braunschweig, Germany; 5 Center of Pharmaceutical Engineering (PVZ), Technische Universität Braunschweig, Franz-Liszt-Straße 35A, 38106 Braunschweig, Germany

**Keywords:** Anti-malaria drugs, Coenzyme A synthesis, Drug discovery, Malaria, 1,3,4-Oxadiazole, Oxazole, Phenotypic screening, *Plasmodium falciparum*, Thieno[2,3-*d*]pyrimidine, Thioether

## Abstract

**Background:**

Malaria is a widespread infectious disease that threatens a large proportion of the population in tropical and subtropical areas. Given the emerging resistance against the current standard anti-malaria chemotherapeutics, the development of alternative drugs is urgently needed. New anti-malarials representing chemotypes unrelated to currently used drugs have an increased potential for displaying novel mechanisms of action and thus exhibit low risk of cross-resistance against established drugs.

**Results:**

Phenotypic screening of a small library (32 kinase-inhibitor analogs) against *Plasmodium falciparum* NF54-*luc* asexual erythrocytic stage parasites identified a diarylthioether structurally unrelated to registered drugs. Hit expansion led to a series in which the most potent congener displayed nanomolar antiparasitic activity (IC_50_ = 39 nM, 3D7 strain). Structure–activity relationship analysis revealed a thieno[2,3-*d*]pyrimidine on one side of the thioether linkage as a prerequisite for antiplasmodial activity. Within the series, the oxazole derivative KuWei173 showed high potency (IC_50_ = 75 nM; 3D7 strain), good solubility in aqueous solvents (1.33 mM), and >100-fold selectivity toward human cell lines. Rescue experiments identified inhibition of the plasmodial coenzyme A synthesis as a possible mode of action for this compound class.

**Conclusions:**

The class of antiplasmodial bishetarylthioethers reported here has been shown to interfere with plasmodial coenzyme A synthesis, a mechanism of action not yet exploited for registered anti-malarial drugs. The oxazole congener KuWei173 displays double-digit nanomolar antiplasmodial activity, selectivity against human cell lines, high drug likeness, and thus represents a promising chemical starting point for further drug development.

**Electronic supplementary material:**

The online version of this article (doi:10.1186/s12936-017-1839-3) contains supplementary material, which is available to authorized users.

## Background

In recent years, the fight against malaria has succeeded in a considerable decrease in clinical episodes, based on the use of insecticide-treated nets (ITNs), indoor residual spraying (IRS), and treatment with combination medicines [1]. Nevertheless, each year there are still hundreds of thousands of fatal outcomes from infection with *Plasmodium* [2]. As resistance against older anti-malarial drugs, such as chloroquine and pyrimethamine, is widespread [3], the World Health Organization (WHO) recommends that artemisinin-based combination therapy (ACT) should be the first-line treatment for *Plasmodium falciparum* malaria [4]. Despite this approach, artemisinin resistance is emerging in South East Asia [5–9]. Broad dissemination of artemisinin resistance to other malaria endemic regions of the world could lead to a dire situation, undoing much of the progress achieved in the last decade. As a consequence, the development of antiplasmodial drugs based on novel chemotypes and new mechanisms of action is urgently required to ensure efficacious treatment of malaria in the future [2].

With a view to identifying novel anti-malarial chemotypes that display novel mechanisms, a phenotypic screening of 32 compounds comprised of kinase inhibitor analogues and other structurally diverse molecules was performed. Viability assays on blood stages of transgenic *P. falciparum* parasites that constitutively express luciferase (*Pf*NF54-*luc),* measuring their bioluminescence in a luciferase assay system were employed. Follow-up studies undertaken with a hit compound revealed structure–activity relationships in the chemical class, suggestive of a specific mechanism of action involving coenzyme A synthesis, which ultimately led to the generation of a new potent and drug-like antiplasmodial molecule.

## Methods

### Luciferase-based viability screening for anti-malarial activity

Asexual erythrocytic stages of transgenic NF54-*luc P. falciparum* were used for the luciferase-based viability assay. These parasites constitutively express high levels of luciferase. The parasites were cultured as described previously [10, 11]. Parasite culture with parasitaemia of 0.5–1% was dispensed in triplicate into white 96-well flat-bottom plates (each well contains 250 µL) (NUNC, Roskilde, Denmark) and incubated in the presence of 3 µM test compounds for 48 h (37 °C, 90% N_2_, 5% CO_2_, and 5% O_2_). 0.01% DMSO was included in the untreated infected RBC cultures as negative control, since the compounds stock initially was diluted in DMSO and each treatment well also contained 0.01% DMSO. Subsequently, 100 µL RPMI1640 media was removed from each well and a 100 µL volume of the Bright-Glo^®^ substrate solution added to each well. The resultant cleavage product of the reaction, light, was measured using a FLUOROSKAN FL luminometer (Thermo), to ascertain viable parasites. Untreated cultures were used as negative controls and to calculate the inhibition rate (0% inhibition of parasite growth). Blasticidin (Sigma-Aldrich, St. Louis, MO, USA), used for selection of transfected parasites, was included as a positive control on each plate and gave >90% inhibition of parasite growth at concentration 2 µg/mL. Results are summarized in Table 1. Compounds with >50% inhibition of viability were rated as actives. Experiments were performed in triplicate and were repeated at least twice.

**Table 1:** Results of biological evaluation

ID	% inhibition, *Pf*NF54-Luc at 3 µM	IC_50_ [µM], *Pf*3D7 + solvent	IC_50_ [µM], *Pf*3D7 + 0.8 mM CoA	IC_50_-ratio (CoA/solvent)	IC_50_ [µM], THP-1	IC_50_ [µM], HEK-293
**3a**	80.8 ± 0.5	0.283 ± 0.073	4.61 ± 0.15	16.3	6.13	>10
**3b**	25.7 ± 2.0 at 30 µM	nd	nd	–	nd	nd
**3c**	37.3 ± 3.7 at 30 µM	nd	nd	–	nd	nd
**3d**	−0.6 ± 5.4	nd	nd	–	nd	nd
**3e**	−7.3 ± 6.9	nd	nd	–	nd	nd
**3f**	1.5 ± 4.3	nd	nd	–	nd	nd
**3g**	−13.1 ± 9.1	nd	nd	–	nd	nd
**3h**	−103.3 ± 4.4	nd	nd	–	nd	nd
**3i**	−5.5 ± 3.8	nd	nd	–	nd	nd
**3j**	−11.0 ± 6.7	nd	nd	–	nd	nd
**3k**	−7.0 ± 1.8	nd	nd	–	nd	nd
**3l**	24.3 ± 3.2	nd	nd	–	nd	nd
**3m**	−8.3 ± 4.1	nd	nd	–	nd	nd
**3n**	−0.82 ± 3.11	nd	nd	–	nd	nd
**3o**	2.28 ± 3.31	nd	nd	–	nd	nd
**3p**	−14.6 ± 12.9	nd	nd	–	nd	nd
**3q**	−12.3 ± 2.2	nd	nd	–	nd	nd
**3r**	7.7 ± 1.8	nd	nd	–	nd	nd
**3s**	−2.8 ± 16.6	nd	nd	–	nd	nd
**6a**	−24.8 ± 13.6	nd	nd	–	nd	nd
**6b**	1.79 ± 2.53	nd	nd	–	nd	nd
**6c**	0.98 ± 1.52	nd	nd	–	nd	nd
**8a**	33.2 ± 6.8	10.5 ± 1.1	16.1 ± 2.2	1.53	nd	>40
**8b**	4.7 ± 2.6	5.37 ± 1.0	39.7 ± 0.7	7.39	nd	>40
**8c**	96.5 ± 1.6	1.26 ± 0.16	2.31 ± 0.17	1.83	>300	>40
**8d**	99.9 ± 0.0	0.0388 ± 0.0010	2.61 ± 0.42	67.3	27.5	>10
**8e**	99.5 ± 0.5	0.0747 ± 0.021	2.58 ± 0.42	34.5	33.6	>10
**8f**	99.7 ± 0.1	0.0958 ± 0.0063	11.2 ± 2.6	117	11	>4
**8g**	95.0 ± 1.1	0.0734 ± 0.0022	0.531 ± 0.007	7.23	16.5	>10
**8h**	−1.9 ± 5.3	nd	nd	–	nd	nd
**8i**	−18.4 ± 10.6	nd	nd	–	nd	nd
**8j**	−36.3 ± 10.6	nd	nd	–	nd	nd
**8k**	98.0 ± 0.2	0.149 ± 0.021	1.06 ± 0.03	7.11	28	>20
BS	99.3 ± 0.7	nd	nd	–	nd	nd
Amb 180,780	nd	0.0370 ± 0.0017	1.51 ± 0.19	40.8	nd	>40
Chloroqu	nd	0.0195 ± 0.0034	0.0271 ± 0.0094	1.39	nd	>40
Artemis	nd	0.00337 ± 0.00065	0.00490 ± 0.0011	1.45	nd	nd
Puromyc	nd	0.0338 ± 0.012	0.0939 ± 0.0016	2.78	nd	0.36
Pantoth	nd	752 ± 82	>10,000	>13.3	nd	nd

*nd* not determined

### Selection and synthesis of additional test molecules

**Fig. 1:**
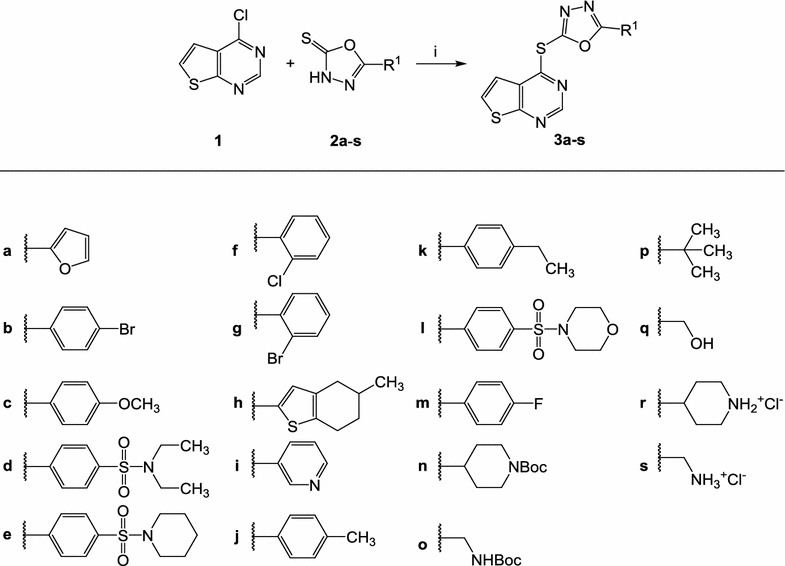
Structures and syntheses of 1,3,4-oxadiazole-containing dihetarylthioethers **3**. Compounds **3a**–**m** were commercially available. Reagents and conditions for **3n**–**q**: *i* triethylamine, DMF or propan-2-ol, 90–120 °C. **3r** and **3s** were synthesized from **3n** and **3o** respectively by cleavage of the protecting group with trifluoroacetic acid in dichloromethane

**Fig. 2:**
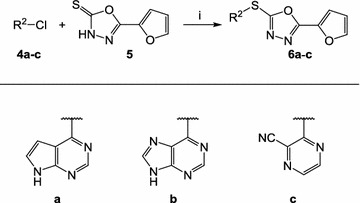
Synthesis of dihetarylthioethers **6a**–**c**. Reagents and conditions: *i* triethylamine, DMF, 100–120 °C

**Fig. 3:**
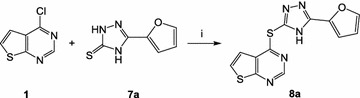
Exemplified synthesis of dihetarylthioether **8a**. Reagents and conditions: *i* triethylamine, DMF, 120 °C

**Fig. 4:**
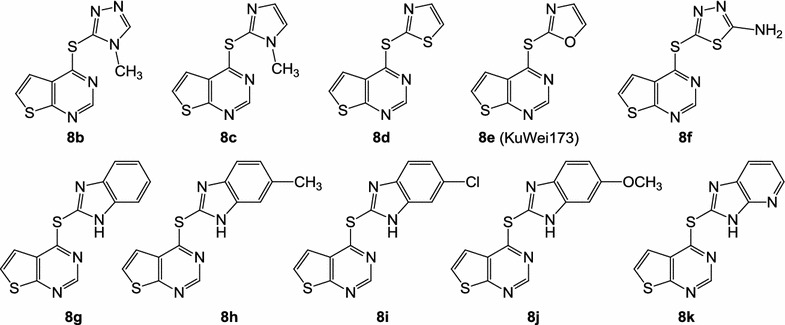
Dihetarylthioethers **8b**–**k** that were synthesized in analogous manner as described for **8a** in Fig. 3. For details of synthesis and characterization, refer to Additional file 2

From the initial screening, compound **3a** (Fig. 1) was identified, representing a chemotype unrelated to established antiplasmodial compounds. As additional test candidates, congeners of **3a** were purchased (**3b**–**3m**, Fig. 1) from Enamine (Enamine Ltd, Kyiv, Ukraine) or synthesized (**3n**–**3s**, Fig. 1; **6a**–**6c**, Fig. 2; **8a**–**8k**, Figs. 3, 4). The syntheses mainly consisted of base-catalyzed nucleophilic aromatic substitution reactions, employing thioxo-substitued heterocycles as nucleophiles and electron-poor heterocycles as substrates, with chloro-substituents as leaving groups. The molecular structure of compound **3p** was corroborated by an X-ray structure analysis (refer to Additional file 1). Compounds **3r** and **3s** were prepared from their precursors **3n** and **3o**, respectively, by cleaving off the Boc protection group using trifluoroacetic acid and precipitating the aminium hydrochlorides with hydrochloric acid from a solution in propan-2-ol and diethyl ether. The structural identity of all new compounds was confirmed by elemental analysis, IR, ^1^H NMR, ^13^C NMR and EI mass spectra. The purity of all synthesized products was >95%, determined by HPLC. Details of synthesis and characterization of all new products are described in Additional file 2. Exemplary ^1^H NMR spectra for compounds **3p**, **6d**, **8d** and **8e** are shown in Additional file 3.

### Prediction of physicochemical parameters

The prediction of physicochemical parameters relevant for drug likeness was performed by computational methods [12]. In the Swiss ADME program package, the topological polar surface area (TPSA) is calculated according to Ertl et al. [13]. Furthermore, the lipophilicity was predicted according to Wildman et al. [14], the solubility was predicted according to Ali et al. [15], and violations of the Pfizer filter for oral bioavailability were assessed according to Lipinski et al. [16]. Results are indicated in Table 2.

**Table 2:** Physicochemical properties of selected test compounds in comparison to Amb180780

ID	M_r_	TPSA	Calc. log P	S_0, exp_ [µM]^a^	Log S_0, exp_	Log S_calc._	Lipinski violations
**3a**	302.33	131.38	3.49	<1.0^b^	<−6.00	−5.30	0
**8c**	248.33	97.14	2.58	1.78 × 10^3 d^	−2.75	−4.06	0
**8d**	251.35	120.45	3.30	36.4^c^	−4.44	−4.78	0
**8e** (Kuwei173)	235.29	105.35	2.83	1.33 × 10^3 c^	−2.88	−3.82	0
**8f**	267.35	159.36	2.28	4.4^c^	−5.36	−5.38	0
**8g**	284.36	108.00	3.72	0.43^d^	−6.37	−5.82	0
**8k**	285.35	120.89	3.11	77.0^c^	−4.11	−5.33	0
Amb180780	265.38	120.45	3.61	nd	nd	−5.94	0

The prediction of physicochemical parameters was performed through Swiss ADME. Calculation of TPSA according to Ertl et al. [13]; lipophilicity prediction according to Wildman et al. [14], solubility prediction according to Ali et al. [15]; Lipinski violations according to Lipinski [16]

*nd* not determined

^a^Determination of thermodynamic solubility in aqueous buffer (pH 7.4) with shake flask method using HPLC

^b^MINIMAL detectable concentration of **3a** by HPLC/UV

^c^Equilibrium after 24 h

^d^Equilibrium after 48 h

### Cytotoxicity assay: human THP-1 cell line

The cytotoxicity against THP-1 cells was determined following a protocol described previously [17] with serial compound dilutions ranging from 200 to 0.1 μM for calculating the IC_50_ values. THP-1 cells were passed into fresh THP-1 culture medium [18]. Cells were harvested by centrifugation (250×*g*, 10′, 4 °C), washed three times with RPMI 1640 medium, and diluted to 8 × 10^5^ cells/mL. The cells were aliquoted in triplicate (125 μL/well) into 96-well flat-bottom plates containing compounds diluted in complete medium (125 μL/well, containing 0.01% DMSO). THP-1 cells in wells containing medium plus 0.01% DMSO served as negative controls. Cells were incubated with test compounds at 37 °C and 5% CO_2_ for 48 h prior to the addition of 10% Alamar Blue (25 μL/well). After an additional 4 h the fluorescence was read (λ_ex_ = 544 nm, λ_em_ = 590 nm) using a microplate reader (Fluoroskan Ascent FL, Thermo Fisher Scientific, Waltham, MA, USA). Results were analysed using Prism 4 (GraphPad Software Inc., La Jolla, CA, USA). Experiments were performed in triplicate and were repeated at least twice.

### Cytotoxicity assay: human HEK-293 cell line

Cytotoxicity against human embryonic kidney (HEK-293) cells was assessed as previously described [19]. Briefly, cells were maintained in DMEM medium supplemented with 10% fetal bovine serum at 37 °C and 5% CO_2_, and routinely split to keep log growth.

Cells were detached using Accutase treatment, then seeded into black clear-bottom 384-wells fluorescence microplates (Greiner), at a cell density of 44,000 cells/mL in 45 µL/well, using a Benchtop liquid dispenser (Thermo Fisher Multidrop). After seeding, the cells were brought back to the incubator and allowed to settle and reattach overnight.

The cells were then exposed to the experimental and reference compounds in full dose–response using three concentrations per log dose (14 points, final concentration range 40 μM–2 nM) and 0.4% final DMSO concentration. Each plate included 5 µM puromycin and 0.4% DMSO as in-plate positive and negative controls, respectively. Plates were incubated at the standard condition above for 72 h. At the end of the incubation the medium in each well was replaced with 1 mM resazurin, and the plates were read using a multilabel microplate reader (PerkinElmer Envision) at 530 nm excitation and 595 nm emission wavelengths after a further 6 h incubation.

Inhibition data was generated using Office Excel 2013 (Microsoft). IC_50_ values were calculated by fitting a four-parameter nonlinear regression model using Prism v.6.0 (Graphpad Software Inc., La Jolla, CA, USA).

### Hypothesis building for antiplasmodial mode of action

**Fig. 5:**
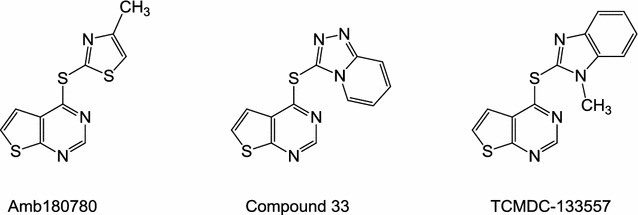
Structures of published antiplasmodial compounds structurally related to series **8**: antiplasmodial compound Amb180780, which interferes with the CoA metabolism; compound 33 from Edlin et al. [26]; TCMDC 133557 from the Tres Cantos Antimalarial Set (TCAMS) [27, 28]

Taking into account the structure–activity relationships obtained from the entire series of compounds, the thieno[2,3-*d*]pyrimidine and its thioether linkage to a second, five-membered heteroaromatic system was assumed as a pharmacophore. This substructure resembles that contained in Amb180780 (Fig. 5), an anti-malarial compound that was previously reported to interfere with plasmodial coenzyme A (CoA) synthesis [19]. A further substructure search in the CAS database of compounds for this pharmacophore revealed an additional 1667 related compounds, however, no other antiplasmodial analogue with an assumed or confirmed mode of action was found.

### Coenzyme A rescue assays

*Plasmodium falciparum* parasites (3D7 strain) were grown in RPMI 1640 supplemented with 25 mM HEPES, 5 mg/mL Albumax II, and 0.37 mM hypoxanthine. Parasites were subjected to two rounds of sorbitol synchronization before undergoing compound treatment. Ring stage parasites were exposed to the experimental compounds alone, or in combination with 0.8 mM coenzyme A (CoA) in 384-wells imaging microplates (CellCarrier, PerkinElmer), as previously described [19]. Chloroquine, artemisinin, puromycin, and also the CoA pathway inhibitors panthenol and Amb180780 were used as reference compounds. Puromycin 5 µM and untreated solvent were used as in-plate positive and negative controls, respectively. Samples were dissolved in DMSO to a final assay DMSO concentration of 0.4%. CoA, chloroquine and panthenol were dissolved in water. All the compounds were tested in full dose–response using three concentrations per log dose (14 points, final concentration range 40 μM–2 nM for the experimental compounds and puromycin; 10 mM–500 nM for panthenol; 10 µM–0.5 nM for artemisinin and chloroquine). All sample and control wells contained the same final amounts of solvents. Plates were incubated for 72 h at 90% N_2_, 5% CO_2_, 5% O_2_, then parasites were stained with 2-(4-amidinophenyl)-1*H*-indole-6-carboxamidine (DAPI), and imaged using an Opera QEHS micro-plate confocal imaging system (PerkinElmer) with 405 nm excitation and 450/50 emission filter, using a 20× water immersion objective. Images were analysed as previously described [20], to obtain normalized % inhibition data, which were then used to calculate IC_50_ values, through a four parameter logistic curve fitting in GraphPad Prism v.6. The experiment was carried out in two independent biological replicates, each consisting of two technical replicates. Dose–response curves are illustrated in the Additional file 4.

### Determination of aqueous solubility

The aqueous solubility was determined using a miniaturized shake-flask-method. In brief, the compound (0.25–1.0 mg) was incubated in a Whatman Mini-Uniprep vial with aqueous phosphate buffer, pH 7.4, (300 µL) in an incubation shaker (IKA^®^ KS 3000 ic control, Staufen, Germany) at 25 °C, 400 rpm. Presence of undissolved compound was checked at 24 and 48 h. After 24, 48, or 72 h of shaking, the filter plunger was punched into the vial and the concentration in the supernatant was determined by HPLC (AUC method, isocratic HPLC, at specific λ_max_ as indicated in the Additional file 2). For the calibration, at least three different dilutions of the compounds from DMSO stock solutions diluted with ACN were quantified. Preparation of buffer pH 7.4: to a solution of Na_2_HPO_4_∙2 H_2_O (290 mg), K_2_HPO_4_ (20 mg) and NaCl (808 mg) in water (ad 100.0 mL), 12% aqueous HCl was added dropwise until the pH reached 7.4. In the case of **3a** the concentration in the supernatant was below the limit of quantification. Therefore, the lowest concentration (1 µM) used for the calibration was indicated as upper limit of solubility in Table 2. Further details of the solubility testing methods are described in Additional file 2, including calibration concentration ranges, composition of chromatographic eluents, and wavelength for measurement of absorbance.

## Results

**Fig. 6:**
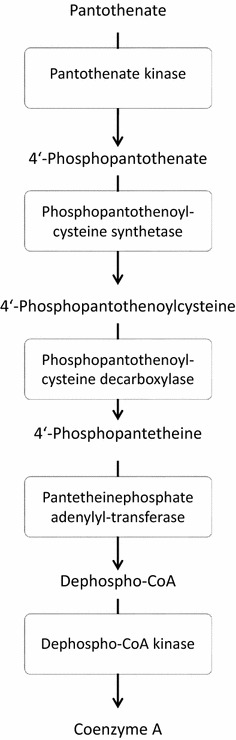
Pathway of CoA biosynthesis. Although the enzymatic steps are conserved, the homology of the plasmodial and human enzymes involved in the pathway is not very high [19, 24]

Testing of the compound library revealed structure **3a** as a hit, which, at a concentration of 3 µM, inhibited the viability of erythrocytic asexual stage *P. falciparum* NF54-*luc* parasites by 80%. Prediction of ADME data by the SwissADME interface predicted **3a** to be only moderately soluble. To generate structure–activity relationships around **3a**, analogues **3b**–**3q** were tested in the same assay. Two congeners with ionizable side chains (**3r**, **3s**) were included in this test run as compounds with predicted improved solubility. All analogues **3** showed, if any, only negligible viability reduction when tested on *Pf*NF54-*luc* parasites at 3 µM (Table 1). Analogues **3b** and **3c** were tested in 30 µM concentration, but even at this high concentration only inhibited the parasites to an extent below 50%, the threshold level for further evaluation. While analogues **6a**–**6c** were also completely devoid of antiplasmodial activity, members of series **8a**–**8k** displayed a broad spectrum of potency. The congeners **8b**, **8h**, **8i**, **8j** were assessed as inactive. Compound **8a**, the analogue most closely related to **3a**, exhibited a medium inhibition of viability (33%). However, the other structures in the series (**8c**–**8g**, **8k**) performed well, producing >95% inhibition of viability in 3 µM concentration. Amb180780 (Fig. 5) is a related structure that was recently identified as an inhibitor of the plasmodial CoA synthesis pathway (Fig. 6) [19]. Considering the structural similarity to Amb180780, the hit structure **3a**, its closely related analogue **8a**, and the most potent compounds **8c**–**8g**, **8k** were evaluated on 3D7 parasites in absence and presence of coenzyme A supplementation. Although **8b** had shown only minor activity against the *Pf*NF54-*luc* strain, the compound was also evaluated in the CoA rescue experiments because of its close structural similarity to the active **8c**. The data from the initial screening on erythrocytes infected with *Pf*NF54-*luc* strain were nicely congruent with the results with 3D7 parasites (Table 1). Test compounds showing >80% inhibition in the initial screening produced submicromolar IC_50_ values against the 3D7 parasites, with **8c** as the only exception (IC_50_ = 1.26 µM). With double-digit nanomolar inhibitory activity, compounds **8d**–**8g** stood out as potent molecules of interest. Supplementation with 0.8 mM CoA clearly rescued the parasites from the inhibitory effects of most of the experimental compounds. A clear reduction in the potency of most compounds was observed in the CoA-supplemented cultures, compared to the solvent controls. With the exception of **8a** and **8c**, all the experimental compounds showed IC_50_ ratios (IC_50_ CoA/IC_50_ solvent) exceeding a threshold of 5×, considered to be significant [21]. The most potent compound was **8d**, with an IC_50_ value of 0.039 ± 0.001 µM in the absence of CoA. Under CoA rescue conditions its IC_50_ shifted to 2.6 ± 0.4 µM (67-fold). KuWei173 (**8e**) was the third most potent compound against *P. falciparum* 3D7, showing IC_50_ values of 0.075 ± 0.002 and 2.6 ± 0.4 µM (34-fold) without and with CoA supplementation, respectively. The largest IC_50_ shift was observed for **8f**, which displayed non-rescue and rescue IC_50_ values of 0.096 ± 0.006 and >11 µM, respectively (117-fold). **8a** and **8c** showed only minor shifts in their IC_50_ values in rescue conditions of ~1.5 and 1.8 respectively. These compounds were not very potent, having non-rescue IC_50_s of 10.5 and 1.3 µM, respectively. All the control compounds showed the expected behaviour in rescue and non-rescue conditions. Chloroquine, artemisinin and puromycin displayed the expected IC_50_ values and were not rescued by coenzyme A supplementation. On the other hand, panthenol and Amb180780 were both rescued by coenzyme A with IC_50_ shifts of 41- and >13-fold, respectively.

Tests of the six most potent antiplasmodial compounds against human cell lines revealed a low degree of cytotoxicity. For example, KuWei173 (**8e**) which displayed an IC_50_ = 75 nM against the 3D7 parasites, showed an IC_50_ of 33.6 µM towards the THP-1 cell line and above 10 µM against HEK-293 cells, corresponding to 450- and >133-fold selectivity, respectively (Table 1).

The aqueous thermodynamic solubility of the compounds showing the highest potency was assessed by a shake flask method at pH 7.4 with HPLC quantification. The hit structure from the initial screening **3a** and the benzimidazole **8g** exhibited poor solubility below 1 µM. While **8d**, **8f** and **8k** were assessed as moderately soluble (solubility between 1 and 100 µM), derivatives **8c** and **8e** (KuWei173) exhibited a remarkable solubility of >1 mM. In the cases of **8c** and **8k** considerable differences of more than one log value were found between predicted and experimentally determined solubility (Table 2).

## Discussion

Given the current emerging resistances against artemisinin-based drugs, discovery and development of anti-malarials acting by hitherto unexploited mechanisms is urgently needed. Starting points for development campaigns in the field of anti-infectives typically are sourced from phenotypic screening. To initiate such a project, a small library of 32 structurally diverse compounds against asexual erythrocytic stages of *Pf*NF54-Luc parasites was screened. Compound **3a** was identified as a hit that showed no resemblance to established anti-malarial compounds and thus offered the possibility of acting via a novel mechanism. However, **3a** displayed poor solubility, an unfavourable property for a drug development (Table 2). For a rational modification of the hit structure, information on structure–activity relationships was needed. The neutral analogues **3b**–**3q** in the initial screening system were therefore tested. The ionizable amines **3r** and **3s** were also included in the assays with the intention to identify analogues with improved solubility (Fig. 1). Compounds **3b**–**3s** all failed to show the desired level of antiplasmodial activity, thus it was speculated that the 2-furyl-1,3,4-oxadiazole system of **3a** would be an important part of the antiplasmodial pharmacophore. This hypothesis was refuted by design, synthesis and testing of the analogues **6a**–**c**, in which the 2-furyl-1,3,4-oxadiazole moiety of **3a** was retained and the thieno[2,3-*d*]pyrimidine was replaced by other heterocycles (Fig. 2). Series **6** was completely inactive. Compound **8a**, an analogue of **3a** in which the oxadiazole oxygen is replaced by nitrogen, was then prepared (Fig. 3). Although **8a** was inferior to **3a**, it still showed at least some (33%) inhibition of the parasites at 3 µM. This result led to the conclusion that the thieno[2,3-*d*]pyrimidine is an indispensable part of the pharmacophore. Consequently, the congeners **8b**–**8k**, in which the thieno[2,3-*d*]pyrimidine element is kept and connected to various five-membered heteroaromatic ring systems by a thioether linkage, were prepared (Fig. 4). Taking into account the results from tests on both *P. falciparum* strains, some conclusions were drawn regarding the contribution of this second heterocyclic element. First, most small heteroaromatic rings were tolerated. However, a methyl substituent adjacent to the thioether linkage led to decreased antiplasmodial activity (analogues **8b** and **8c**). Benzoannulation at the site opposite to the thioether linkage was tolerated and resulted in the very potent derivative **8g**. Since **8g** shows only submicromolar aqueous solubility, the aza analogue **8k** was designed, which showed a slightly decreased antiplasmodial activity but remarkably improved solubility by more than two orders of magnitude (77 µM). The introduction of substituents at the five-position of the benzimidazole element completely shut down the antiplasmodial activity, as was exemplified by **8h**, **8i**, and **8j**. It appears that in the binding pocket of an as yet unidentified target protein the available space around this position is restricted.

The benchmark for compounds suitable for drug discovery and clinical candidates is an aqueous solubility of >60 µg/mL [22], which translates to a 200 µM solution for molecules with a molecular mass of ~300 g mol^−1^. Of the compounds showing antiplasmodial activity in this study, compounds **8c** and **8e** (KuWei173) met this demand, exhibiting >1 mM solubility. Since KuWei173 (**8e**) shows 17-fold higher antiplasmodial activity it clearly represents the most promising result from the present study.

It has been shown that besides aqueous solubility, the topological polar surface area (TPSA) is a well-suited parameter for the oral bioavailability prediction of drugs. As a rule of thumb, molecules displaying less than 60 Å^2^ TPSA are absorbed to an extent of >90%, while compounds with more than 140 Å^2^ TPSA are absorbed by ≤10%. KuWei173 (**8e**) has a calculated TPSA of 105 Å^2^ and, therefore, appears more promising both in terms of solubility and permeability than the closely related thiazole analogues **3d** and Amb180780, which both show TPSA values ~120 Å^2^ (Table 2).

For a rational structure-based drug development process with KuWei173 (**8e**) as the lead compound, the identification of a biological target in the parasite would be of great value. We previously reported that Amb180780 (Fig. 5), a compound structurally related to **8e**, inhibits *Pf*3D7 parasites by interfering with the CoA synthesis pathway [19].

*Plasmodium falciparum* is capable of CoA de-novo synthesis and is thus independent of CoA synthesis by the host [23]. Although the CoA synthesis pathway is highly conserved among eukaryotes (Fig. 6), the homology of the five enzymes catalyzing the transformation of pantothenate to CoA between *Plasmodium* and mammalian species is not high [19, 24]. Consequently, the CoA synthesis pathway was suggested as a potential target for antiplasmodial drugs [19, 25]. Considering the structural similarity to Amb180780, a similar mode of action for members of the series **8** was postulated. Upon evaluation of **8a**–**8g, 8k** and **3a** for antiplasmodial activity in the absence and presence of coenzyme A, we noted that CoA supplementation rescued the parasites from inhibition by the compounds, with the exception of **8a** and **8c**. The results corroborated the hypothesis that inhibition of CoA synthesis was a mechanism associated with the observed antiplasmodial activity for this series. The two outliers **8a** and **8c** are less active compared to other molecules, and may act by other mechanisms than CoA synthesis inhibition. Low potency alone, however, does not explain lack of rescue by CoA, because **8b**, the second least potent compound (IC_50_ = 6.4 ± 4.3 µM in non-rescue conditions) was significantly rescued by CoA supplementation to an IC_50_ of ~40 µM, corresponding to a ~sevenfold shift in activity.

In terms of solubility and antiplasmodial activity in vitro, KuWei173 (**8e**) appears as the most interesting compound resulting from the project reported here. Follow-up studies are now warranted with KuWei173 (**8e**) to assess parameters including metabolic stability and in vivo efficacy.

While searching for analogues of series **8** the two antiplasmodial thieno[2,3-*d*]pyrimidines compound 33 [26] and TCMDC-124514 [27] (Fig. 5) were also encountered. The latter was designated as a “quality chemical starting point” [27] among the 13,533 hit compounds of the Tres Cantos Anti-malarial Set, which resulted from the screening of nearly 2 million compounds from GSK [28]. Indeed, TCMDC-124514 differs only by one methyl group from the compound **8g** presented here. To the best of our knowledge, neither a systematic structure optimization study nor a mechanism of antiplasmodial action has been published for TCMDC-124514. It is tempting to speculate that on the one hand TCMDC-124514 may also act by inhibition of CoA synthesis, and that on the other hand the methyl substituent at the ring nitrogen may negatively influence the antiplasmodial activity, considering the results with **8b** and **8c**. In contrast, compound 33, published by Edlin et al. [26], was the best compound in a series that was systematically developed from a HTS hit. In a *Plasmodium berghei* infected mouse model, compound 33 injected at 50 mg/kg subcutaneously over 4 days produced a 34% drop in parasitaemia, but failed to prolong the life-span of infected animals. The authors rated compound 33 as promising for further follow-up through optimization of ADME properties. In this respect KuWei173 (**8e**) can be considered as optimized analogue of compound 33 at least in terms of solubility and potency, although it has not yet been studied for metabolic stability in vitro or in vivo malaria models.

For further structure-guided optimization of this compound class, it will be necessary to identify which of the enzymes involved in CoA synthesis constitutes the eventual target. A recent study using the same rescue approach used here, but with additional CoA pathway intermediates found that addition of pantethine, dephospho CoA and CoA all rescued the activity of Amb180780 to more than 75%, pointing at the enzymes phosphopantothenoylcysteine synthetase or phosphopantothenoylcysteine decarboxylase as the putative targets for Amb180780 [29]. For similar studies aiming at corroborating the putative targets of the new compound class presented here, the readily soluble and potent inhibitor KuWei173 (**8e**) is now available.

## Conclusion

A phenotypic screening campaign towards new antiplasmodial drugs was performed by testing a compound library on *Pf*NF54-*luc* parasites. Modification of hit structure **3a** led to the simple dihetarylthioether KuWei173 (**8e**) which shows high viability inhibition on asexual erythrocytic *P. falciparum* parasites and good water solubility. Studies with CoA supplementation showed that KuWei173 most probably acts by inhibition of the plasmodial CoA synthesis pathway. Cytotoxicity testing further revealed a considerable safety margin towards human cell lines. Taken together, KuWei173 (**8e**) appears to be a good lead structure for further development and a useful tool to study plasmodial CoA biosynthesis.

## Additional files

**Additional file 1.** X-ray structure analysis of test compound **3p**.

**Additional file 2.** Synthesis procedures and characterization of test compounds **3n**–**3s**, **6a**–**6c**, **8a**–**8k** and details of solubility testing.

**Additional file 3.** ^1^H NMR spectra of test compounds **3p**, **6d**, **8d**, **8e**.

**Additional file 4.** Dose–response plots for test compounds and reference drugs showing parasite viability (3D7) in absence and presence of coenzyme A.

## Abbreviations

ACT: artemisinin-based combination therapy
ADME: absorption, distribution, metabolism, excretion
AUC: area under the curve
CAS: chemical abstracts service
CoA: coenzyme A
DAPI: 2-(4-amidinophenyl)-1*H*-indole-6-carboxamidine
DMEM: Dulbecco’s modified Eagle’s medium
DMSO: dimethyl sulfoxide
EI: electron impact
GSK: GlaxoSmithKline
HEK: human embryonic kidney
HEPES: 4-(2-hydroxyethyl)-1-piperazineethanesulfonic acid
HPLC: high performance liquid chromatography
HTS: high throughput screening
IC_50_: concentration for 50% inhibition
IR: infra-red
IRS: indoor residual spraying
ITN: insecticide-treated net
NMR: nuclear magnetic resonance
RBC: red blood cell
RPMI: Roswell Park Memorial Institute
THP-1: a human monocytic leukemia cell line
TPSA: topological polar surface area
WHO: World Health Organization
