# Supplementary material for: Antiplasmodial dihetarylthioethers target the coenzyme A synthesis pathway in Plasmodium falciparum erythrocytic stages
Source: Malar J. 2017 May 15;16:192. doi: 10.1186/s12936-017-1839-3 (PMC5430599; doi:10.1186/s12936-017-1839-3)
Supplement: Supplementary file 1 — Additional file 1. X-ray structure analysis of test compound 3p. [file 12936_2017_1839_MOESM1_ESM.pdf]

Additional file 1: X-ray crystallographic structure of **3p**

Experimental: An irregular pale yellow prism ca. 0.4 × 0.2 × 0.2 mm was mounted on a glass fibre in inert oil and transferred to the cold gas stream of the diffractometer (Oxford Diffraction Xcalibur E). Data were recorded at 100 K using monochromated Mo *K*α radiation. Absorption corrections were based on multi-scans. The structure was refined anisotropically on F^2 using the program SHELXL-97 (G. M. Sheldrick, University of Göttingen, Germany). Methyls were refined as idealized rigid groups allowed to rotate but not tip; other hydrogens were included using a riding model starting from calculated positions.

Complete crystallographic data have been deposited with the Cambridge Crystallographic Data Centre as supplementary publication no. CCDC-1526841. Copies of the data can be obtained free of charge from www.ccdc.cam.ac.uk/data_request/cif.

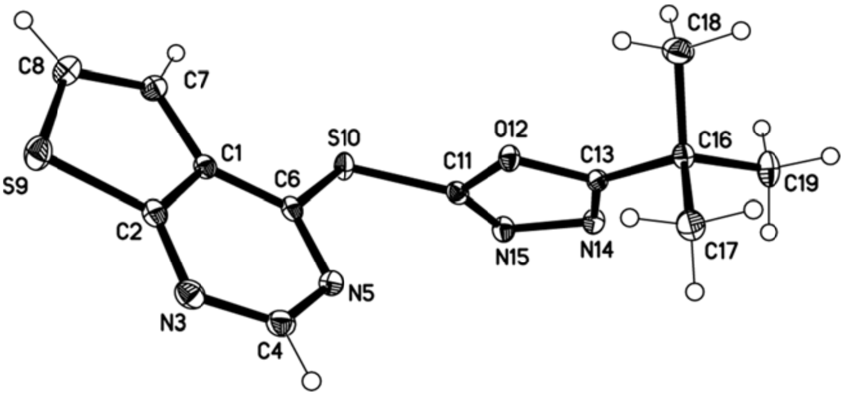

Additional file 1: Figure 1**:** X-ray crystallographic structure of **3p**.

Additional file 1: Table 1**:** Crystal data and structure refinement of **3p**.

Empirical formula	$C_{12}H_{12}N_4OS_2$
Formula weight	292.38
Temperature	100(2) K
Wavelength	0.71073 Å
Crystal system	monoclinic
Space group	C2/c
Unit cell dimensions	a = 16.8835(8) Å α = 90°
	b = 11.3347(4) Å β = 106.216(5)°
	c = 14.2477(7) Å γ = 90°
Volume	2618.1(2) Å^3
Z	8
Density (calculated)	1.484 Mg/m^3
Absorption coefficient	0.403 mm^{-1}
F(000)	1216
Crystal size	0.4 x 0.2 x 0.2 mm^3

Theta range for data collection	2.19 to 31.10°
Index ranges	-24<=h<=22, -16<=k<=16, -20<=l<=20
Reflections collected	34143
Independent reflections	3969 [R(int) = 0.0487]
Completeness to Theta = 30.00°	98.0%
Absorption correction	Semi-empirical from equivalents
Max. and min. transmission	1.00000 and 0.94434
Refinement method	Full-matrix least-squares on F^2
Data / restraints / parameters	3969 / 0 / 175
Goodness-of-fit on F^2	1.069
Final R indices [I>2sigma(I)]	R1 = 0.0332, wR2 = 0.0748
R Indices (all data)	R1 = 0.0431, wR2 = 0.0805
Largest diff. peak and hole	0.476 and -0.319 e.Å^{-3}

Additional file 1: Table 2: Atomic coordinates (x 10^4) and equivalent isotropic displacement parameters (Å^2 x 10^3). U(eq) is defined as one third of the trace of the orthogonalized U_{ij} tensor.[a]

	x	y	z	U(eq)
C(1)	5813.6(7)	-356.0(10)	4360.8(9)	11.1(2)
C(2)	5114.5(8)	-979.9(11)	3811.9(9)	12.3(2)
N(3)	4417.4(7)	-476.4(10)	3272.2(8)	15.1(2)
C(4)	4436.9(8)	696.2(11)	3278.5(9)	14.8(2)
N(5)	5050.6(7)	1404.8(9)	3779.5(8)	13.6(2)
C(6)	5724.3(7)	870.8(10)	4320.4(9)	11.1(2)
C(7)	6476.6(8)	-1120.7(11)	4840.8(9)	13.7(2)
C(8)	6269.9(8)	-2269.2(11)	4651.9(10)	16.9(3)
S(9)	5279.5(2)	-2484.5(3)	3905.2(2)	17.3(1)
S(10)	6530.3(2)	1732.9(3)	5069.0(2)	13.5(1)
C(11)	6414.7(7)	3049.2(11)	4419.9(9)	11.6(2)
O(12)	6475.5(5)	3073.3(7)	3484.3(6)	11.9(2)
C(13)	6511.2(7)	4257.7(10)	3315.2(9)	10.6(2)
N(14)	6484.8(7)	4881.7(9)	4065.4(8)	13.4(2)
N(15)	6412.1(7)	4079.5(9)	4794.5(8)	14.1(2)
C(16)	6552.6(7)	4666.6(10)	2331.2(9)	11.6(2)
C(17)	5705.1(8)	4466.0(12)	1595.2(9)	16.3(2)
C(18)	7215.3(8)	3966.6(12)	2019.5(10)	18.5(3)
C(19)	6774.9(9)	5979.0(11)	2401.1(10)	17.9(3)

[a] Values in brackets correspond to the standard deviation with regard to the last decimal digit.

Additional file 1: Table 3: Bond lengths [Å][a]

C(1)-C(6)	1.3981(16)	S(10)-C(11)	1.7375(12)
C(1)-C(2)	1.4103(17)	C(11)-N(15)	1.2846(16)
C(1)-C(7)	1.4300(17)	C(11)-O(12)	1.3656(14)
C(2)-N(3)	1.3405(16)	O(12)-C(13)	1.3682(14)

C(2)-S(9)	1.7272(13)	C(13)-N(14)	1.2928(16)
N(3)-C(4)	1.3295(17)	C(13)-C(16)	1.4967(17)
C(4)-N(5)	1.3472(16)	N(14)-N(15)	1.4115(14)
N(5)-C(6)	1.3284(16)	C(16)-C(19)	1.5306(17)
C(6)-S(10)	1.7702(12)	C(16)-C(18)	1.5349(18)
C(7)-C(8)	1.3552(18)	C(16)-C(17)	1.5365(17)
C(8)-S(9)	1.7303(14)		

[a] Values in brackets correspond to the standard deviation with regard to the last decimal digit.

Additional file 1: Table 4: Bond angles [°][a]

C(6)-C(1)-C(2)	114.33(11)	N(15)-C(11)-O(12)	113.41(10)
C(6)-C(1)-C(7)	133.12(11)	N(15)-C(11)-S(10)	124.92(10)
C(2)-C(1)-C(7)	112.55(11)	O(12)-C(11)-S(10)	120.59(9)
N(3)-C(2)-C(1)	124.71(11)	C(11)-O(12)-C(13)	102.16(9)
N(3)-C(2)-S(9)	124.15(9)	N(14)-C(13)-O(12)	112.20(10)
C(1)-C(2)-S(9)	111.13(9)	N(14)-C(13)-C(16)	128.79(11)
C(4)-N(3)-C(2)	113.93(11)	O(12)-C(13)-C(16)	118.99(10)
N(3)-C(4)-N(5)	127.84(12)	C(13)-N(14)-N(15)	106.63(10)
C(6)-N(5)-C(4)	116.30(11)	C(11)-N(15)-N(14)	105.59(10)
N(5)-C(6)-C(1)	122.83(11)	C(13)-C(16)-C(19)	108.35(10)
N(5)-C(6)-S(10)	119.19(9)	C(13)-C(16)-C(18)	109.85(10)
C(1)-C(6)-S(10)	117.92(9)	C(19)-C(16)-C(18)	109.63(10)
C(8)-C(7)-C(1)	111.32(11)	C(13)-C(16)-C(17)	108.37(10)
C(7)-C(8)-S(9)	114.12(10)	C(19)-C(16)-C(17)	110.67(10)
C(2)-S(9)-C(8)	90.88(6)	C(18)-C(16)-C(17)	109.95(10)
C(11)-S(10)-C(6)	101.54(6)	N(15)-C(11)-O(12)	113.41(10)

[a] Values in brackets correspond to the standard deviation with regard to the last decimal digit.

Additional file 1: Table 5: Torsion angles [°][a]

C(6)-C(1)-C(2)-N(3)	1.24(18)	N(5)-C(6)-S(10)-C(11)	-28.96(11)
C(7)-C(1)-C(2)-N(3)	-178.20(11)	C(1)-C(6)-S(10)-C(11)	153.85(9)
C(6)-C(1)-C(2)-S(9)	-179.94(9)	C(6)-S(10)-C(11)-N(15)	132.85(11)
C(7)-C(1)-C(2)-S(9)	0.63(13)	C(6)-S(10)-C(11)-O(12)	-59.80(10)
C(1)-C(2)-N(3)-C(4)	1.06(18)	N(15)-C(11)-O(12)-C(13)	0.00(13)
S(9)-C(2)-N(3)-C(4)	-177.62(10)	S(10)-C(11)-O(12)-C(13)	-168.71(9)
C(2)-N(3)-C(4)-N(5)	-2.46(19)	C(11)-O(12)-C(13)-N(14)	0.67(13)
N(3)-C(4)-N(5)-C(6)	1.26(19)	C(11)-O(12)-C(13)-C(16)	-177.89(10)
C(4)-N(5)-C(6)-C(1)	1.49(17)	O(12)-C(13)-N(14)-N(15)	-1.04(13)
C(4)-N(5)-C(6)-S(10)	-175.56(9)	C(16)-C(13)-N(14)-N(15)	177.35(11)
C(2)-C(1)-C(6)-N(5)	-2.57(17)	O(12)-C(11)-N(15)-N(14)	-0.59(14)
C(7)-C(1)-C(6)-N(5)	176.71(12)	S(10)-C(11)-N(15)-N(14)	167.55(9)
C(2)-C(1)-C(6)-S(10)	174.51(8)	C(13)-N(14)-N(15)-C(11)	0.98(13)
C(7)-C(1)-C(6)-S(10)	-6.21(19)	N(14)-C(13)-C(16)-C(19)	13.87(17)

C(6)-C(1)-C(7)-C(8)	-179.43(13)	O(12)-C(13)-C(16)-C(19)	-167.85(10)
C(2)-C(1)-C(7)-C(8)	-0.14(15)	N(14)-C(13)-C(16)-C(18)	133.59(13)
C(1)-C(7)-C(8)-S(9)	-0.42(14)	O(12)-C(13)-C(16)-C(18)	-48.12(14)
N(3)-C(2)-S(9)-C(8)	178.11(11)	N(14)-C(13)-C(16)-C(17)	-106.26(14)
C(1)-C(2)-S(9)-C(8)	-0.72(10)	O(12)-C(13)-C(16)-C(17)	72.02(13)
C(7)-C(8)-S(9)-C(2)	0.67(11)		

[a] Values in brackets correspond to the standard deviation with regard to the last decimal digit.
