# Supplementary material for: Antiplasmodial dihetarylthioethers target the coenzyme A synthesis pathway in Plasmodium falciparum erythrocytic stages
Source: Malar J. 2017 May 15;16:192. doi: 10.1186/s12936-017-1839-3 (PMC5430599; doi:10.1186/s12936-017-1839-3)
Supplement: Supplementary file 2 — Additional file 2. Synthesis procedures and characterization of test compounds 3n–3s, 6a–6c, 8a–8k and details of solubility testing. [file 12936_2017_1839_MOESM2_ESM.pdf]

Additional file 2: Synthesis procedures and characterization of test compounds

Apparatus and Materials

Congeners of **3a** were purchased (**3b-3m**) from Enamine (Enamine Ltd, Kyiv, Ukraine) or synthesized (**3n-3s**, **6a-6c**, **8a-8k**). Starting materials for syntheses were purchased from commercial suppliers and were used without further purification. Microwave-assisted syntheses were performed in sealed vials (10 mL) in a mono mode microwave device (CEM Discover SP; CEM GmbH, Kamp-Lintford, Germany). Dichloromethane was dried by distillation from CaH_2. Melting points were determined on an electric variable heater (Electrothermal IA9200, Bibby Scientific, Stone, UK) in open glass capillaries and are uncorrected. IR spectra were recorded as KBr disks on a Thermo Nicolet FT-IR 200 (Thermo Nicolet, Madison, WI, USA). ^{1}H-NMR spectra and ^{13}C-NMR spectra were recorded on BrukerAvance DRX-400 and BrukerAvance II-600 instruments (Bruker Corporation, Billerica, MA, USA) (at the NMR laboratories of the Chemical Institutes of the Technische Universität Braunschweig). Chemical shifts were recorded as δ values in ppm and are referenced to an internal standard tetramethylsilane. Signals in ^{13}C spectra were assigned based on the result of ^{13}C DEPT135 experiments. Elemental analyses were determined on a CE Instruments FlashEA 1112 elemental analyzer (Thermo Quest, San Jose, CA, USA). Mass spectra were recorded on a Finnigan-MAT 95 (Thermo Finnigan MAT, Bremen, Germany). Accurate measurements were conducted according to the peak match method using perfluorokerosene (PFK) as an internal mass reference. (EI) MS: ionization energy 70 eV (Department of Mass Spectrometry of the Chemical Institutes of the Technische Universität Braunschweig).TLC: Polygram Sil G/UV_{254} (Macherey-Nagel, Düren, Germany), 40 mm × 80 mm, visualization by UV illumination (254 and 366 nm). Purity was determined by HPLC using isocratic and gradient elution performed on Merck Hitachi Elite LaChrom systems (Hitachi High Technologies Inc., San Jose, CA, USA): pump L-2130, autosampler L-2200, diode array detector L-2450 (isocratic elution) or UV detector L-2400 (gradient elution), organizer box L-2000; column, Merck LiChroCART 125-4, LiChrosphere 100, RP 18, 5 µm (Merck, Darmstadt, Germany); flow rate 1.000 mL/min; detection wavelength: 254 and 280 nm (isocratic elution) and 254 nm (gradient elution); AUC, % method; time of detection 15 min (isocratic elution) or 20 min (gradient elution), retention time (t_{M+S}); dead time (t_M) related to DMSO. For isocratic runs, mixtures of ACN and water or mixtures of ACN and buffer were

used. For all gradient runs, mixtures of ACN and water were used (gradient: 0–2 min: 10% ACN, 2–12 min: 10% → 90% ACN, linear, 12–20 min 90% ACN). Preparation of H_2O + $(Et_3NH)_2SO_4$buffer (pH 2.7) for isocratic HPLC: triethylamine (20.0 mL) and sodium hydroxide (242 mg) were dissolved in water to 1 L. The solution was adjusted to pH 2.7 by addition of sulfuric acid. All compounds which were biologically tested were of >95% purity. Absorption maxima (λ_{max}) were extracted from the spectra recorded by the DAD in the HPLC peak maxima in isocratic runs (software, EZ Chrom Elite Client/server, version 3.1.3., Scientific Software Inc., Pleasanton, CA, USA).

General procedure 1 for the synthesis of **3n**, **3o**, **3q**, **6a**-**c**, **8a**, **8b**, **8d**, **8e**, **8h**-**j** (GP1)

In a microwave device vial the appropriate chloro-substituted heteroaromatic compound (1.0 equivalent) is dissolved in the indicated solvent (e.g. DMF, propan-2-ol) (2-3 mL) together with the appropriate thione compound (1.1-1.2 equivalents) and a base (e.g. triethylamine, caesium carbonate) (1.1-1.2 equivalents). After sealing the microwave vial with a sealing cap equipped with a Teflon inlay, the mixture is stirred at 90-140 °C under microwave irradiation (conditions: 100-200 W, 5 min ramp time, 20-150 min reaction time, maximum pressure, 145 psi). Afterwards the reaction mixture is poured onto ice (10 g) and the precipitated solid is filtered off. The resulting residue is further purified by recrystallization or by column chromatography over silica gel.

General procedure 2 for the synthesis of **8c**, **8g**, **8k** (GP2)

A stirred solution of 4-chlorothieno[2,3-*d*]pyrimidine (1.0 equivalent) in the indicated solvent (e.g. DMF, propan-2-ol) (2-4 mL) together with the appropriate thione compound (1.0-1.2 equivalents) and triethylamine (1.1-1.2 equivalents) is heated to 83-120 °C for 22-48 h. Afterwards the reaction mixture is poured onto ice (10 g) and the precipitated solid is filtered off. The resulting residue is further purified by recrystallization or column chromatography over silica gel.

General procedure for the synthesis of **3r** and **3s** (GP3)

To a stirred solution of the Boc-protected starting material **3n** or **3o** (1.0 equivalent) in dried dichloromethane (10-20 mL) under argon or nitrogen atmosphere, trifluoroacetic acid (10-32 equivalents) is added at room temperature. The reaction is stopped after 18-25 h and the solvent is evaporated under reduced pressure. The resulting residue is dissolved in a

minimum amount of propan-2-ol (0.5 mL). Hydrochloric acid solution (37%) (2-5 drops) is added until a precipitate is formed. Then diethyl ether is added (15-20 mL) and the mixture is heated under reflux for 30-60 min. After cooling to room temperature the solid is filtered off and further purified by recrystallization.

tert-Butyl 4-(5-(thieno[2,3-*d*]pyrimidin-4-ylthio)-1,3,4-oxadiazol-2-yl)piperidine-1-carboxylate (**3n**)

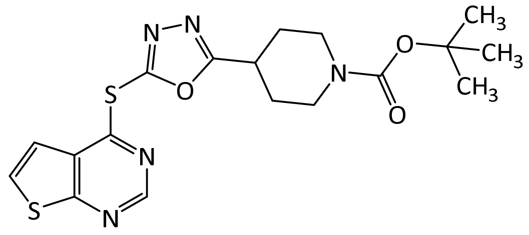

According to GP1, a solution of 4-chlorothieno[2,3-*d*]pyrimidine (150 mg, 0.880 mmol), tert-butyl 4-(5-thioxo-4,5-dihydro-1,3,4-oxadiazol-2-yl)piperidine-1-carboxylate[1] (285 mg, 0.999 mmol) and triethylamine (0.14 L, 1.0 mmol) in DMF (2.5 mL) was stirred for 20 min at 90 °C under microwave irradiation (100 W). Crystallization from methanol yielded slightly yellow crystals (184 mg, 0.439 mmol, 50%).

Mp.: 139-140 °C; IR (KBr): $\tilde{\nu}$ [cm^{-1}] = 1691 (C=O); ^{1}H-NMR: (600 MHz, DMSO-d_6): δ [ppm] = 1.40 (s, 9H, $C(CH_3)_3$), 1.55-1.69 (m, 2H, CH_2), 1.97-2.08 (m, 2H, CH_2), 2.99 (s, 2H, CH_2), 3.90 (d, J = 12.9 Hz, 2H, CH_2), 7.63 (d, J = 6.0 Hz, 1H, CH (thiophene)), 8.14 (d, J = 6.0 Hz, 1H, CH (thiophene)), 8.84 (s, 1H, CH (pyrimidine)), the signal for piperidyl-CH overlaps with the water-peak between 3.31 and 3.36 ppm and cannot be integrated properly; ^{13}C-NMR (151 MHz, DMSO-d_6) δ [ppm] = 28.04 (3C, CH_3), 28.39 (2C), 41.89 (2C) (CH_2), 32.40, 118.70, 130.44, 152.52 (CH), 78.83, 127.44, 130.44, 153.84, 156.17, 158.48, 167.08, 172.58 (C); $C_{18}H_{21}N_5O_3S_2$ (419.52): calc. C 51.53, H 5.05, N 16.69, found C 51.45, H 4.99, N 16.62; MS (EI): m/z (%) = 419.1 [$M^{+\cdot}$] (2), 152.1 [$M^{+\cdot}$-267] (100); HPLC (isocratic): 99.1% at 254 nm and 99.7% at 280 nm, t_{M+S} = 7.10 min, t_M(DMSO) = 1.06 min (ACN/water = 50:50), λ_{max} [nm] = 231, 284; HPLC (gradient): 99.3%, t_{M+S} = 12.10 min, t_M(DMSO) = 1.28 min.

tert-Butyl ((5-(thieno[2,3-*d*]pyrimidin-4-ylthio)-1,3,4-oxadiazol-2-yl)methyl)carbamate (**3o**)

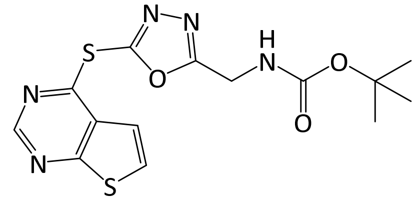

According to GP1, a solution of 4-chlorothieno[2,3-*d*]pyrimidine (150 mg, 0.880 mmol), *tert*-butyl ((5-thioxo-4,5-dihydro-1,3,4-oxadiazol-2-yl)methyl)carbamate[1] (231 mg, 0.999 mmol) and triethylamine (0.14 mL, 1.0 mmol) in DMF (2.5 mL) was stirred for 40 min at 90 °C under microwave irradiation (100 W). Crystallization from ethanol yielded beige crystals (123 mg, 0.337 mmol, 38%).

Mp.: 153-154 °C; IR (KBr): $\tilde{\nu}$ [cm^{-1}] = 3369 (N-H), 1689 (C=O); ^{1}H-NMR: (600 MHz, DMSO-d_6): δ [ppm] = 1.37 (s, 9H, $C(CH_3)_3$), 4.47 (d, *J* = 6.1 Hz, 2H, CH_2), 7.64 (d, *J* = 6.0 Hz, 1H, CH (thiophene)), 7.69 (t, *J* = 6.1 Hz, 1H, NH), 8.14 (d, *J* = 6.0 Hz, 1H, CH (thiophene)), 8.83 (s, 1H, CH (pyrimidine)); ^{13}C-NMR (151 MHz, DMSO-d_6) δ [ppm] = 28.08 (3C, CH_3), 35.56 (CH_2), 118.76, 130.52, 152.47 (CH), 78.78, 127.56, 155.52, 156.52, 158.28, 167.15, 168.86 (C); $C_{14}H_{15}N_5O_3S_2$ (365.43): calc. C 46.02, H 4.14, N 19.17, found C 46.07, H 4.21, N 19.12; MS (EI): m/z (%): 209.0 (100); HPLC (isocratic): 98.7% at 254 nm and 97.4% at 280 nm, t_{M+S} = 3.09 min, t_M(DMSO) = 1.06 min (ACN/water = 50:50), λ_{max} [nm] = 231, 284; HPLC (gradient): 96.6%, t_{M+S} =10.26 min, t_M(DMSO) = 1.28 min.

2-(*tert*-Butyl)-5-(thieno[2,3-*d*]pyrimidine-4-ylthio)-1,3,4-oxadiazole (**3p**)

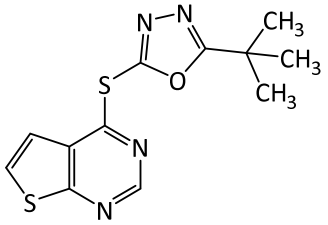

According to GP1, a solution of 5-*tert*-butyl-1,3,4-oxadiazole-2(3*H*)-thione[2] (158 mg, 0.999 mmol), 4-chlorothieno[2,3-*d*]pyrimidine (150 mg, 0.884 mmol) and triethylamine (0.14 mL, 1.0 mmol) in a mixture of propan-2-ol (2 mL) and DMF (0.5 mL) was stirred for 40 min at 120 °C under microwave irradiation (200 W). Purification by column chromatography (EA/PE 1:4) yielded a slightly yellow solid (97 mg, 0.33 mmol, 38%).

Crystallization from ethyl acetate yielded crystals which were analyzed by X-ray crystallographic methods (crystallographic data is summarized in Additional file 1).

Mp.: 129-130 °C; IR (KBr): $\tilde{\nu}$ [cm^{-1}] = 3069 (=C-H), 2972 (-C-H); ^{1}H-NMR (600 MHz, $CDCl_3$) δ [ppm] = 1.47 (s, 9H, CH_3), 7.34 (d, J = 6.0 Hz, 1H, CH (thiophene), 7.64 (d, J = 6.0 Hz, CH (thiophene)), 8.76 (s, 1H, CH (pyrimidine)); ^{13}C-NMR: (151 MHz, $CDCl_3$) δ [ppm] = 27.77 (CH_3), 118.05, 128.21, 152.37 (CH), 32.72, 127.61, 156.16, 158.78, 167.38, 177.32 (C); $C_{12}H_{12}N_4OS_2$ (292.38): calc. C 49.30, H 4.14, N 19.16, found C 49.69, H 3.97, N 19.12; MS (EI): m/z (%) = 292.1 $[M]^{+\cdot}$ (11), 209.0 $[M^{+\cdot}-83]$ (100); HPLC (isocratic): 99.6% at 254 nm and 99.8% at 280 nm, t_{M+S} = 5.29 min, t_M (DMSO) = 1.06 min (ACN/water = 50:50), λ_{max} [nm] = 234, 284; HPLC (gradient): t_{M+S} = 11.60 min, t_M (DMSO) = 1.28 min.

5-Hydroxymethyl-2-(thieno[2,3-*d*]pyrimidine-4-ylthio)-1,3,4-oxadiazole (**3q**)

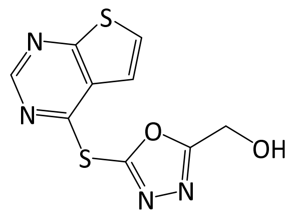

According to GP1, a suspension of 5-hydroxymethyl-1,3,4-oxadiazole-2(3*H*)-thione[3] (195 mg, 1.48 mmol), 4-chlorothieno[2,3-*d*]pyrimidine (204 mg, 1.20 mmol) and triethylamine (0.21 mL, 1.5 mmol) in propan-2-ol (4 mL) was stirred for 30 min at 120 °C under microwave irradiation (200 W). Purification by column chromatography (PE/EA 5:3 → 1:1 → 1:2 → 0:1) and subsequent crystallization from ethyl acetate yielded a colorless solid (42 mg, 0.16 mmol, 13%).

Mp.: 156-157 °C; IR (KBr): $\tilde{\nu}$ [cm^{-1}] = 3304 (s, O-H), 3097 (m, =C-H), 2929 (w, -C-H) ; ^{1}H-NMR (600 MHz, DMSO-d_6) δ [ppm] = 4.75 (d, J = 6.4 Hz, 2H, CH_2), 6.07 (t, J = 6.4 Hz, 1H, OH), 7.66 (d, J = 6.0 Hz, 1H, CH (thiophene)), 8.15 (d, J = 6.0 Hz, 1H, CH (thiophene)), 8.85 (s, 1H, CH (pyrimidine)); ^{13}C-NMR: (151 MHz, DMSO-d_6) δ [ppm] = 53.87 (CH_2), 118.75, 130.49, 152.54 (CH), 127.52, 156.63, 167.11, 170.26 (C); $C_9H_6N_4O_2S_2$ (266.29): calc. C 40.59, H 2.27, N 21.04, found C 40.59, H 2.02, N 20.77; MS (EI): m/z (%): 266.0 $[M]^{+\cdot}$ (10), 209.0 $[M^{+\cdot}-57]$ (100); HPLC (isocratic): 96.2% at 254 nm and 95.7% at 280 nm, t_{M+S} = 2.98 min, t_M (DMSO) = 1.06 min (ACN/water = 25:75), λ_{max} [nm] = 236, 286; HPLC (gradient): 97.0% at 254 nm, t_{M+S} = 7.51 min, t_M (DMSO) = 1.26 min.

4-(5-(Thieno[2,3-*d*]pyrimidin-4-ylthio)-1,3,4-oxadiazol-2-yl)piperidin-1-ium chloride (**3r**)

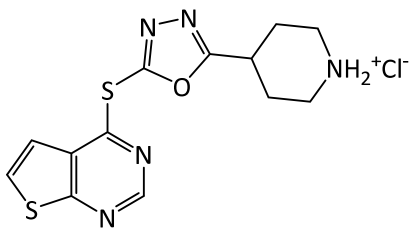

According to GP3, a solution of *tert*-butyl 4-(5-(thieno[2,3-*d*]pyrimidin-4-ylthio)-1,3,4-oxadiazol-2-yl)piperidine-1-carboxylate (**3n**) (150 mg, 0.358 mmol) and trifluoroacetic acid (870 µL, 11.3 mmol) in dichloromethane (20 mL) was stirred for 18 h at room temperature under argon atmosphere. Crystallization from ethanol yielded a slightly beige solid (47 mg, 0.13 mmol, 37%).

Mp.: 217-219 °C (decomp.); IR (KBr): $\tilde{\nu}$ [cm^{-1}] = 3400 (m, br, N-H), 2851 (m,-C-H); ^{1}H-NMR (600 MHz, DMSO-d_6) δ [ppm] = 1.92-2.06 (m, 2H, CH_2), 2.17–2.28 (m, 2H, CH_2), 3.06 (t, J = 12.3 Hz, 2H, CH_2), 3.24-3.33 (m, 2H, CH_2), 3.46-3.61 (m, 1H, CH), 7.66 (d, J = 6.0 Hz, 1H, CH (thiophene)), 8.16 (d, J = 6.0 Hz, 1H, CH (thiophene)), 8.84 (s, 1H, CH (pyrimidine)), 8.92 (s, 1H, NH_2^+), 9.07 (s, 1H, NH_2^+); ^{13}C-NMR: (151 MHz, DMSO-d_6) δ [ppm] = 25.22 (2C), 41.86 (2C) (CH_2), 30.28, 118.74, 130.53, 152.50 (CH), 127.47, 156.51, 158.45, 167.10, 171.68 (C); MS (EI): m/z (%) = 319.0 $[M_{free\ base}]^{+\cdot}$ (3), 210.0 $[M^{+\cdot}$-109] (100); HRMS (EI): calc. for $C_{13}H_{13}N_5OS_2$ 319.05560, found 319.05490; HPLC (isocratic): 99.5% at 254 nm and 99.1% at 280 nm, t_{M+S} = 3.71 min, t_M (DMSO) = 1.06 min (ACN/buffer$_{pH\ 2.7}$ = 15:85), λ_{max} [nm] = 232, 286.

(5-(Thieno[2,3-*d*]pyrimidin-4-ylthio)-1,3,4-oxadiazol-2-yl)methanaminium chloride (**3s**)

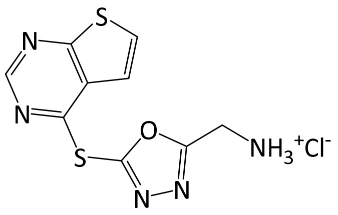

According to GP3, a solution of *tert*-butyl ((5-(thieno[2,3-*d*]pyrimidin-4-ylthio)-1,3,4-oxadiazol-2-yl)methyl)carbamate (**3o**) (90 mg, 0.25 mmol) and trifluoroacetic acid (0.57 mL, 2.5 mol) in dichloromethane (10 mL) was stirred under nitrogen atmosphere at room temperature for 25 h. The trifluoroacetic acid was added in three equal portions (each 0.19 mL) at the beginning of the reaction, after 3 h and after 18 h reaction time.

Crystallization from ethanol/petroleum ether (1:2, 15 mL) yielded a slightly beige solid (20 mg, 0.066 mmol, 27%).

Mp.: 176-180 °C (decomp.); IR (KBr): $\tilde{\nu}$ [cm^{-1}] = 3400 (s, br, N-H) ; ^{1}H-NMR (600 MHz, DMSO-*d*$_6$) δ [ppm] = 4.56 (s, 2H, CH_2), 7.69 (d, *J* = 6.0 Hz, 1H, CH (thiophene)), 8.18 (d, *J* = 6.0 Hz, 1H, CH (thiophene)), 8.85 (s, 1H, CH (pyrimidine)), 8.93 (br, s, 3H, NH_3^+, exchangable); ^{13}C-NMR: (151 MHz, DMSO-*d*$_6$) δ [ppm] = 33.65 (CH_2), 118.86, 130.75, 152.48 (CH), 127.81, 157.73, 157.85, 165.22, 167.29 (C); MS (EI): *m/z* (%) = 265.0 $[M_{free\ base}]^{+\cdot}$ (9), 209.0 $[M^{+\cdot}$-54] (100); HRMS (EI): calc. for $C_9H_7N_5OS_2$ 265.00865, found 265.00915 HPLC (isocratic): 98.8% at 254 nm and 98.6% at 280 nm, t_{M+S} = 3.73 min, t_M (DMSO) = 1.06 min (ACN/buffer$_{pH\ 2.7}$ = 10:90), λ_{max} [nm] = 242, 286.

5-(Furan-2-yl)-2-((7*H*-pyrrolo[2,3-*d*]pyrimidine-4-yl)thio)- 1,3,4-oxadiazole (**6a**)

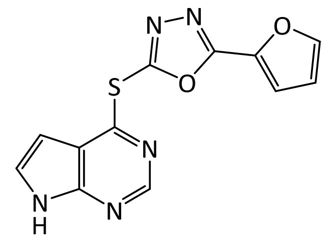

According to GP1, a solution of 5-(furan-2-yl)-1,3,4-oxadiazole-2(3*H*)-thione[4] (118 mg, 0.702 mmol), 4-chloro-7*H*-pyrrolo[2,3-*d*]pyrimidine (92 mg, 0.60 mmol) and triethylamine (97 µL, 0.70 mmol) in DMF (2 mL) was stirred for 30 min at 120 °C under microwave irradiation (200 W). Crystallization from ethanol (70%) yielded brown crystals (112 mg, 0.393 mmol, 65%).

Mp.: 178-182 °C; IR (KBr): $\tilde{\nu}$ [cm^{-1}] = 3414 (N-H); ^{1}H-NMR (400 MHz, DMSO-*d*$_6$) δ [ppm] = 6.48 (dd, *J* = 3.6, 1.7 Hz, 1H, CH (pyrrole)), 6.84 (dd, *J* = 3.6, 1.8 Hz, 1H, CH (furan)), 7.50 (dd, *J* = 3.5, 0.8 Hz, 1H, CH (furan)), 7.67 (dd, *J* = 3.6, 2.4 Hz, 1H, CH (pyrrole)), 8.12 (dd, *J* = 1.8, 0.7 Hz, 1H, CH (furan)), 8.56 (s, 1H, CH (pyrimidine)), 12.53 (s, 1H, NH); ^{13}C-NMR: (101 MHz, DMSO-*d*$_6$) δ [ppm] = 97.94, 112.88, 115.92, 128.15, 147.71, 150.47 (CH), 115.51, 137.95, 150.32, 153.77, 156.64, 160.20 (C); $C_{12}H_7N_5O_2S$ (285.28): calc. C 50.52, H 2.47, N 24.55, found C 50.50, H 2.46, N 24.29; MS (EI): m/z (%) = 285.1 $[M]^{+\cdot}$ (6) 192.0 $[M^{+\cdot}$-93] (100); HPLC (isocratic): 99.5% at 254 nm and 99.6% at 280 nm, t_{M+S} = 7.15 min, t_M (DMSO) = 1.06 min (ACN/water = 50:50), λ_{max} [nm] = 225, 241, 284; HPLC (gradient): 98.7%, t_{M+S} = 12.16 min, t_M (DMSO) = 1.28 min.

6-[(5-Furan-2-yl)-1,3,4-oxadiazole-2-ylthio]purine (**6b**)

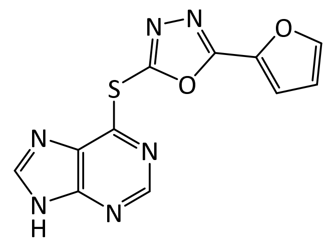

According to GP1, a solution of 5-(furan-2-yl)-1,3,4-oxadiazole-2(3*H*)-thione[4] (168 mg, 0.999 mmol) and 6-chloropurine (136 mg, 0.880 mmol) and triethylamine (0.14 mL, 1.0 mmol) in DMF (2.5 mL) was stirred for 20 min at 100 °C and subsequently another 20 min at 120 °C under microwave irradiation (100 W). Crystallization from ethanol (70%) yielded a slightly brown solid (130 mg, 0.454 mmol, 52%).

Mp.: 176-177 °C; IR (KBr): $\tilde{\nu}$ [cm^{-1}] = 3427 (br, N-H); ^{1}H-NMR (600 MHz, DMSO-d_6) δ [ppm] = 6.85 (dd, *J* = 3.6, 1.8 Hz, 1H, CH (furan)), 7.49 (dd, *J* = 3.6, 0.7 Hz, 1H, CH (furan)), 8.12 (dd, *J* = 1.9, 0.7 Hz, 1H, CH (furan)), 8.58 (s, 1H, CH (purine)), 8.70 (s, 1H, CH (purine)), 13.82 (s, 1H, NH); ^{13}C-NMR: (151 MHz, DMSO-d_6) δ [ppm] = 112.94, 115.89, 147.71, 151.72 (CH) 138.11, 145.24, 156.03, 160.19 (C); $C_{11}H_6N_6O_2S$ (286.27): calc. C 46.15, H 2.11, N 29.36, found C 46.01, H 2.09, N 29.09; MS (EI): m/z (%) = 286.0 $[M]^{+\cdot}$ (6), 193.0 [$M^{+\cdot}$-93] (100); HPLC (isocratic): 98.4% at 254 nm and 98.8% at 280 nm, t_{M+S} = 4.43 min, t_M (DMSO) = 1.06 min (ACN/water = 30:70), λ_{max} [nm] = 283, 229; HPLC (gradient): 95.7%, t_{M+S} = 7.77 min, t_M (DMSO) = 1.28 min.

3-[5-(Furan-2-yl)-1,3,4-oxadiazole-2yl-thio]pyrazine-2-carbonitrile (**6c**)

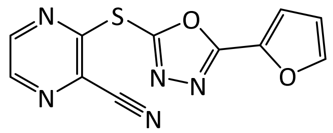

According to GP1, a solution of 5-(furan-2-yl)-1,3,4-oxadiazole-2(3*H*)-thione[4] (168 mg, 0.999 mmol), 3-chloropyrazine-2-carbonitrile (123 mg, 0.881 mmol) and triethylamine (0.14 mL, 1.0 mmol) in DMF (2.5 mL) was stirred for 40 min at 120 °C under microwave irradiation (100 W). Crystallization from ethanol (70%) yielded red-brown crystals (124 mg, 0.457 mmol, 52%).

Mp.: 149-150 °C; IR (KBr): $\tilde{\nu}$ [cm^{-1}] = 2235 (w, C≡N); ^{1}H-NMR: (600 MHz, DMSO-d_6) δ [ppm] = 6.85 (dd, *J* = 3.7, 1.8 Hz, 1H, CH (Furan)), 7.49 (dd, *J* = 3.7, 0.8 Hz, 1H, CH (Furan)), 8.13 (dd, *J* = 1.8, 0.8 Hz, 1H, CH (furan)), 8.80 (d, *J* = 2.4 Hz, 1H, CH (pyrazine)), 8.82 (d, *J* = 2.4 Hz, 1H, CH (pyrazine)); ^{13}C-NMR: (151 MHz, DMSO-d_6) δ [ppm] = 112.98, 116.13, 143.92, 147.88, 148.06 (CH), 114.53, 128.81, 137.80, 154.60, 156.28, 160.18 (C); $C_{11}H_5N_5O_2S$ (271.25): calc. C 48.71, H 1.86, N 25.82, found C 48.58, H 1.82, N 25.75; MS (EI): m/z (%) = 271.0 [M]$^{+\cdot}$ (34), 95.0 [M$^{+\cdot}$-176] (100); HPLC (isocratic): 99.2% at 254 nm and 99.4% at 280 nm, t_{M+S} = 4.45 min, t_M(DMSO) = 1.06 min (ACN/water = 40:60), λ_{max} [nm] = 261, 275, 297; HPLC (gradient): 98.9%, t_{M+S} =10.02 min, t_M (DMSO) = 1.28 min.

<u>5-(Furan-2-yl)-3-(thieno[2,3-*d*]pyrimidine-4-ylthio)-1,2,4-triazole (**8a**)</u>

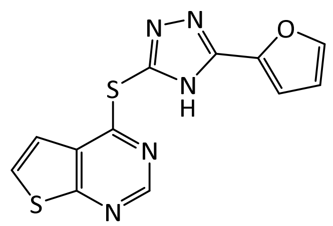

According to GP1, a solution of 4-chlorothieno[2,3-*d*]pyrimidine (102 mg, 0.601 mmol), 5-(furan-2-yl)-1,2,4-triazole-3(4*H*)-thione (104 mg, 0.622 mmol) and triethylamine (84 µL, 0.61 mmol) in DMF (2 mL) was stirred for 40 min at 120 °C under microwave irradiation (200 W). Crystallization from methanol/water (1:1) yielded a beige solid (130 mg, 0.431 mmol, 72%).

Mp.: 167–168 °C; IR (KBr): $\tilde{\nu}$ [cm^{-1}] = 3430 (w, br, N-H), 1512 (s); ^{1}H-NMR: (600 MHz, DMSO-d_6): δ [ppm] = 6.66, 6.75 (2s, br, 2H, arom. H), 7.00, 7.18 (2s, br 1H), 7.38 – 7.71 (m, 2H, arom. H), 7.77 – 8.18 (m, 4H, arom. H), 8.72-8.89 (m,4 Hz, 1H), 15.05, 15.19 (2s, br, 2H, NH, exchangable), the spectrum shows two tautomeric forms of **8a** (ratio approx. 1:0.4); ^{13}C-NMR: (151 MHz, DMSO-d_6) δ [ppm] = 109.44, 111.68, 112.36, 118.79, 129.38, 129.63, 129.71, 130.01, 133.77, 133.86, 145.40, 152.54 (CH), 118.72, 127.34, 142.01, 142.54, 143.95, 145.87, 148.82, 151.82, 156.28, 161.72, 166.49, 166.72 (C), the spectrum shows two tautomeric forms of **8a**; $C_{12}H_7N_5OS_2$ (301.34): calc. C 47.83, H 2.34, N 23.24, found C 47.90, H 2.10, N 22.85; MS (EI): m/z (%) = 301.0 [M]$^{+\cdot}$ (100), 180.0 [M$^{+\cdot}$-121] (35), 135.0 [M$^{+\cdot}$-166] (34); HPLC (isocratic): 99.9% at 254 nm and 99.7% at 280 nm, t_{M+S} = 3.32 min, t_M(DMSO) =

1.06 min (ACN/buffer$_{pH\ 2.7}$ = 35:65), λ_{max} [nm] = 229, 263; HPLC (gradient): 97.1% at 254 nm, t_{M+S} = 8.83 min, t_M(DMSO) = 1.28 min.

4-Methyl-3-(thieno[2,3-*d*]pyrimidine-4-ylthio)-1,2,4-triazole (**8b**)

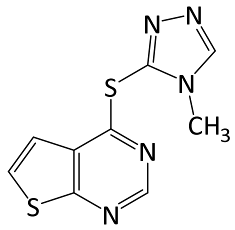

According to GP1, a solution of 4-chlorothieno[2,3-*d*]pyrimidine (102 mg, 0.601 mmol), 3-mercapto-4-methyl-1,2,4-triazole (145 mg, 1.26 mmol) and triethylamine (166 µL, 1.20 mmol) in DMF (3 mL) was stirred for 60 min at 120 °C under microwave irradiation (200 W). Crystallization from ethyl acetate yielded slightly yellow crystals (22 mg, 0.088 mmol, 15%).

Mp.: 201–203 °C; IR (KBr): $\tilde{\nu}$ [cm^{-1}] = 3092 (w, =C-H), 2958 (w, -C-H), 1506 (s); ^{1}H-NMR: (600 MHz, DMSO-d_6): δ [ppm] = 3.64 (s, 3H, CH_3), 7.63 (d, *J* = 6.0 Hz, 1H, CH (thiophene)), 8.11 (d, *J* = 6.0 Hz, 1H, CH (thiophene)), 8.77 (s, 1H, arom. H), 8.92 (s, 1H, arom. H); ^{13}C-NMR: (151 MHz, DMSO-d_6) δ [ppm] = 31.47 (CH_3), 118.68, 129.84, 148.09, 152.54 (CH), 126.90, 141.98, 160.02, 166.62 (C); $C_9H_7N_5S_2$ (249.31): calc. C 43.36, H 2.83, N 28.09, found C 43.46, H 2.74, N 27.81; MS (EI): m/z (%) = 249.0 $[M]^{+\cdot}$ (100), 135.0 [$M^{+\cdot}$-114] (44); HPLC (isocratic): 99.9% at 254 nm and 99.9% at 280 nm, t_{M+S} = 4.25 min, t_M(DMSO) = 1.06 min (ACN/water = 20:80), λ_{max} [nm] = 228, 287; HPLC (gradient): 99.1% at 254 nm, t_{M+S} = 7.29 min, t_M(DMSO) = 1.28 min.

4-[1-Methyl-1*H*-imidazol-2-yl)thio]thieno[2,3-*d*]pyrimidine (**8c**)

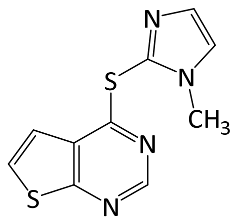

According to GP2, a solution of 2-mercapto-1-methylimidazole (114 mg, 0.999 mmol), 4-chlorothieno[2,3-*d*]pyrimidine (150 mg, 0.880 mmol) and triethylamine (0.14 mL, 1.0 mmol)

in propan-2-ol (4 mL) was stirred for 48 h under reflux. Crystallization from ethanol (50%) yielded slightly yellow needles (70 mg, 0.28 mmol, 32%).

Mp.: 158-159 °C; IR (KBr): $\tilde{\nu}$ [cm^{-1}] = 3101, 3074, 3056, 3027 (=C-H), 2949 (-C-H) ; ^{1}H-NMR (600 MHz, DMSO-d_6) δ [ppm] = δ 3.64 (s, 3H, CH_3), 7.18 (d, *J* = 1.3 Hz, 1H, CH (imidazole)), 7.32 (d, *J* = 6.0 Hz, 1H, CH (thiophene)), 7.61 (d, *J* = 1.2 Hz, 1H, CH (imidazole)), 8.05 (d, *J* = 6.0 Hz, 1H, CH (thiophene)), 8.76 (s, 1H, CH (pyrimidine)); ^{13}C-NMR: (151 MHz, DMSO-d_6) δ [ppm] = 33.61 (CH_3), 118.56, 126.20, 129.36, 130.11, 152.49 (CH), 126.64, 131.44, 162.17, 166.56 (C); $C_{10}H_8N_4S_2$ (248.32): calc. C 48.37, H 3.25, N 22.56, found C 48.32, H 3.18, N 22.54; MS (EI): m/z (%) = 248.0 $[M]^{+\cdot}$ (99), 215.1 $[M^{+\cdot}$-33] (100); HPLC (isocratic): 98.9% at 254 nm and 98.8% at 280 nm, t_{M+S} = 5.91 min, t_M (DMSO) = 1.06 min (ACN/buffer$_{pH\ 2.7}$ = 16:84), λ_{max} [nm] = 239, 285.

2-(Thieno[2,3-*d*]pyrimidine-4-ylthio)-1,3-thiazole (**8d**)

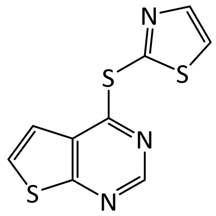

According to GP1, a solution of 4-chlorothieno[2,3-*d*]pyrimidine (119 mg, 0.702 mmol), 1,3-thiazole-2-thiol (73 mg, 0.62 mmol) and triethylamine (0.10 mL, 0.72 mmol) in DMF (2 mL) was stirred for 30 min at 120 °C under microwave irradiation (200 W). Crystallization from methanol yielded brown needles (112 mg, 0.446 mmol, 72%).

Mp.: 132–133 °C; IR (KBr): $\tilde{\nu}$ [cm^{-1}] = 3100 (w), 1512 (s); ^{1}H-NMR: (400 MHz, DMSO-d_6): δ [ppm] = 7.59 (d, *J* = 6.0 Hz, 1H, CH (thiophene)), 8.06 (d, *J* = 3.3 Hz, 1H, CH (thiazole)), 8.09-8.14 (m, 2H, CH (thiophene), CH (thiazole)), 8.93 (s, 1H, CH (pyrimidine)); ^{13}C-NMR: (101 MHz, DMSO-d_6) δ [ppm] = 118.62, 126.03, 129.78, 143.40, 152.13 (CH), 126.87, 152.70, 159.76, 166.46 (C); $C_9H_5N_3S_3$ (251.34): calc. C 43.01, H 2.01, N 16.72, found C 43.27, H 1.94, N 16.72; MS (EI): m/z (%) = 251.0 $[M]^{+\cdot}$ (100), 135.0 $[M^{+\cdot}$-116] (43); HPLC (isocratic): 98.7% at 254 nm and 99.2% at 280 nm, t_{M+S} = 5.07 min, t_M(DMSO) = 1.06 min (ACN/water = 45:55), λ_{max} [nm] = 231, 293; HPLC (gradient): 98.8% at 254 nm, t_{M+S} = 10.95 min, t_M(DMSO) = 1.28 min.

2-(Thieno[2,3-*d*]pyrimidine-4-ylthio)-1,3-oxazole (Kuwei 173, **8e**)

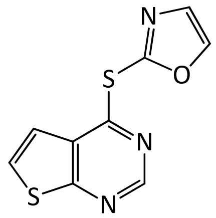

According to GP1, a solution of 4-chlorothieno[2,3-*d*]pyrimidine (110 mg, 0.649 mmol), 1,3-oxazole-2-thiol (59 mg, 0.58 mmol) and triethylamine (90 µL, 0.65 mmol) in DMF (2 mL) was stirred for 60 min at 120 °C under microwave irradiation (200 W). Crystallization from methanol (50%) yielded a beige solid (28 mg, 0.12 mmol, 21%).

Mp.: 89–90 °C; IR (KBr): $\tilde{\nu}$ [cm^{-1}] = 1512 (s); ^{1}H-NMR: (600 MHz, DMSO-d_6): δ [ppm] = 7.49 (d, *J* = 6.0 Hz, 1H, CH (thiophene)), 7.58 (d, *J* = 0.9 Hz, 1H, CH (oxazole)), 8.11 (d, *J* = 6.0 Hz, 1H, CH (thiophene)), 8.52 (d, *J* = 0.9 Hz, 1H, CH (oxazole)), 8.82 (s, 1H, CH(pyrimidine)); ^{13}C-NMR: (151 MHz, DMSO-d_6) δ [ppm] = 118.60, 130.09, 130.28, 145.38, 152.53, (CH), 127.21, 150.25, 160.12, 166.86 (C); $C_9H_5N_3OS_2$ (235.29): calc. C 45.95, H 2.14, N 17.86, found C 46.23, H 2.19, N 17.81; MS (EI): m/z (%) = 235.0 $[M]^{+\cdot}$ (100), 135.0 $[M^{+\cdot}$-100] (82); HPLC (isocratic): 97.2% at 254 nm and 97.2% at 280 nm, t_{M+S} = 4.69 min, t_M(DMSO) = 1.06 min (ACN/water = 35:65), λ_{max} [nm] = 231, 286; HPLC (gradient): 95.9% at 254 nm, t_{M+S} = 9.48 min, t_M(DMSO) = 1.28 min.

5-(Thieno[2,3-*d*]pyrimidine-4-ylthio)-1,3,4-thiadiazole-2-amine (**8f**)

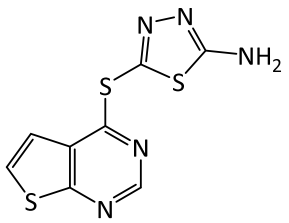

A suspension of 5-amino-1,3,4-thiadiazole-2-thiol (102 mg, 0.766 mmol), 4-chlorothieno[2,3-*d*]pyrimidine (102 mg, 0.601 mmol) and potassium carbonate (101 mg, 0.731 mmol) in DMF (5 mL) was stirred for 18 h at room temperature and for 5 h at 50 °C. Subsequently the reaction mixture was poured onto ice (10 g). The precipitate thus formed was filtered off and suspended in a mixture of ethyl acetate and methanol (5:1, 36 mL). The mixture was

heated for 1 h under reflux and the product was filtered off when still hot. Thus the product was obtained as a beige solid (57 mg, 0.21 mmol, 36%).

Mp.: 219-220 °C (decomp.); IR (KBr): $\tilde{\nu}$ [cm^{-1}] = 3257 (m, br, N-H); ^{1}H-NMR (400 MHz, DMSO-*d*$_6$) δ [ppm] = 7.59 (d, *J* = 6.0 Hz, 1H, CH (thiophene)), 7.74 (s, 2H, NH_2), 8.09 (d, *J* = 6.0 Hz, 1H, CH (thiophene)), 8.89 (s, 1H, CH (pyrimidine)); ^{13}C-NMR: (101 MHz, DMSO-*d*$_6$) δ [ppm] = 118.59, 129.70, 152.33 (CH), 126.85, 140.46, 160.32, 166.49, 173.23 (C); $C_8H_5N_5S_3$ (267.34): calc. C 35.94, H 1.89, N 26.20, found C 35.95, H 1.74, N 26.11; MS (EI): m/z (%) = 267.0 $[M]^{+\cdot}$ (20), 225.0 $[M-42]^{+\cdot}$ (100); HPLC (isocratic): 99.7% at 254 nm and 99.7% at 280 nm, t_{M+S} = 8.11 min, t_M(DMSO) = 1.06 min (ACN/water = 20:80), λ_{max} [nm] = 231, 289; HPLC (gradient): 99.2%, t_{M+S} = 8.20 min, t_M(DMSO) = 1.28 min.

4-[(Benzimidazole-2-yl)thio]thieno[2,3-*d*]pyrimidine (**8g**)

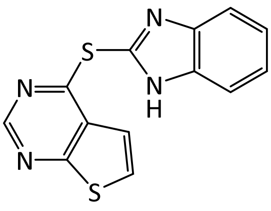

According to GP2, a solution of benzimidazole-2-thiol (105 mg, 0.699 mmol), 4-chlorothieno[2,3-*d*]pyrimidine (102 mg, 0.601 mmol) and triethylamine (0.10 mL, 0.72 mmol) in DMF (2.5 mL) was stirred for 22 h at 120 °C. Crystallization from acetone yielded a beige solid (35 mg, 0.12 mmol, 18%).

Mp.: 217-220 °C; IR (KBr): $\tilde{\nu}$ [cm^{-1}] = 3434 (m, br, N-H); ^{1}H-NMR (600 MHz, DMSO-*d*$_6$) δ [ppm] = 7.29 (br s, 2H, arom. H), 7.49 (d, *J* = 6.0 Hz, 1H, CH (thiophene)), 7.50-7.82 (m, 2H, arom. H), 8.08 (d, *J* = 6.0 Hz, 1H, CH (thiophene)), 8.82 (s, 1H, CH (pyrimidine)), 13.25 (s, 1H, NH); ^{13}C-NMR: (151 MHz, DMSO-*d*$_6$) δ [ppm] =118.82, 129.68, 152.58 (CH), 111.60, 119.26, 122.11, 123.57, 127.35, 135.66, 139.73, 143.59, 161.04, 166.67; $C_{13}H_8N_4S_2$ (284.36): calc. C 54.91, H 2.84, N 19.70, found C 54.65, H 2.82, N 19.67; MS (EI): m/z (%) = 284.0 $[M]^{+\cdot}$ (100), 226.0 $[M^{+\cdot}-58]$ (62); HPLC (isocratic): 99.4% at 254 nm and 99.7% at 280 nm, t_{M+S} = 4.81 min, t_M(DMSO) = 1.06 min (ACN/water = 40:60), λ_{max} [nm] = 235, 289; HPLC (gradient): 99.9%, t_{M+S} = 10.7 min, t_M(DMSO) = 1.28 min.

4-[(5-Methyl-1*H*-benzimidazole-2-yl)thio]thieno[2,3-*d*]pyrimidine (**8h**)

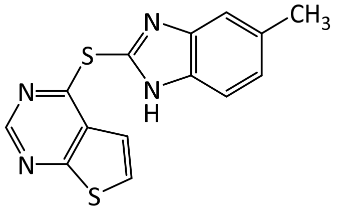

According to GP1, a solution of 5-methylbenzimidazole-2-thiol (115 mg, 0.700 mmol), 4-chlorothieno[2,3-*d*]pyrimidine (102 mg, 0.601 mmol) and caesium carbonate (228 mg, 0.700 mmol) in DMF (2 mL) was stirred for 45 min at 140 °C under microwave irradiation (200 W). Crystallization from ethanol yielded a beige solid (52 mg, 0.17 mmol, 29%).

Mp.: 243-244 °C; IR (KBr): $\tilde{\nu}$ [cm^{-1}] = 3430 (N-H); ^{1}H-NMR (600 MHz, DMSO-d_6) δ [ppm] = 2.29 (s, 3H, CH_3), 2.39 (s, 3H, CH_3), 6.93-6.96 (m, 1H, arom. H), 6.97–7.04 (m, 2H, arom. H), 7.08–7.17 (m, 2H, arom. H), 7.20 (d, *J* = 8.1 Hz, 1H, arom. H), 7.37-7.45 (m, 2H, arom. H), 8.03-8.11 (m, 2H, arom. H), 9.27 (2s, 2H, CH (pyrimidine)), 13.24 (s, 2H, NH); ^{13}C-NMR: (151 MHz, DMSO-d_6) δ [ppm] = 20.86, 20.92 (CH_3), 109.79, 110.18, 110.29, 110.64, 120.91, 121.00, 123.74, 124.98, 129.32, 129.45, 153.40, 153.47 (CH), 127.01, 127.24, 129.54, 130.64, 131.79, 132.57, 132.79, 133.80, 150.23, 150.26, 168.23, 168.31, 170.73, 170.75 (C), the spectra show two tautomeric forms of **8h** (ratio approx. 1:1); $C_{14}H_{10}N_4S_2$ (298.38): calc. C 56.36, H 3.38, N 18.78, found C 56.43, H 3.32, N 18.69; MS (EI): m/z (%) = 298.1 $[M]^{+\cdot}$ (100), 240.1 $[M^{+\cdot}-58]$ (95); HPLC (isocratic): 99.9% at 254 nm and 99.9% at 280 nm, t_{M+S} = 4.19 min, t_M(DMSO) = 1.06 min (ACN/water = 40:60), λ_{max} [nm] = 227, 234, 304; HPLC (gradient): 97.5% at 254 nm, t_{M+S} = 10.36 min, t_M(DMSO) = 1.28 min.

4-[(5-Chloro-1*H*-benzimidazole-2-yl)thio]thieno[2,3-*d*]pyrimidine (**8i**)

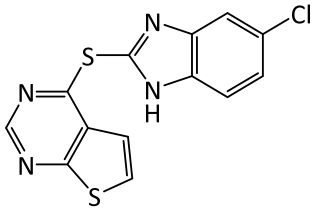

According to GP1, a solution of 5-chlorobenzimidazole-2-thiol (130 mg, 0.704 mmol), 4-chlorothieno[2,3-*d*]pyrimidine (102 mg, 0.601 mmol) and diisopropylethylamine (0.12 mL, 0.69 mmol) in propan-2-ol (2 mL) was stirred for 150 min at 120 °C under microwave

irradiation (200 W). Crystallization from ethanol yielded a beige solid (53 mg, 0.17 mmol, 28%).

Mp.: 188–190 °C; IR (KBr): $\tilde{\nu}$ [cm^{-1}] = 3430 (N-H); ^{1}H-NMR (600 MHz, DMSO-d_6) δ [ppm] = 7.32 (br s, 1H, CH (benzene)), 7.51 (d, J = 6.0 Hz, 1H, CH (thiophene)), 7.54–7.87 (m, 2H, CH (benzene)), 8.09 (d, J = 6.0 Hz, 1H, CH (thiophene)), 8.83 (s, 1H, CH (pyrimidine)), 13.44 (s, 1H, NH); ^{13}C-NMR: (151 MHz, DMSO-d_6) δ [ppm] = 118.84, 129.82, 152.57 (CH), 111.39, 113.08, 118.53, 119.70, 122.56, 123.76, 127.45, 130.72, 160.56, 166.74 (C); $C_{13}H_7ClN_4S_2$ (318.80): calc. C 48.98, H 2.21, N 17.57, found C 48.90, H 2.03, N 17.40; MS (EI): m/z (%) = 318.0 [$M^{+\cdot}$] (100), 260.0 [$M^{+\cdot}$-58] (76); HPLC (isocratic): 96.7% at 254 nm and 97.9% at 280 nm, t_{M+S} = 4.79 min, t_M(DMSO) = 1.06 min (ACN/buffer$_{pH\ 2.7}$ = 50:50), λ_{max} [nm] = 237, 301.

<u>4-[(5-Methoxy-1*H*-benzimidazole-2-yl)thio]thieno[2,3-*d*]pyrimidine (**8j**)</u>

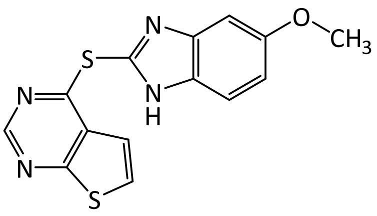

According to GP1, a solution of 5-methoxybenzimidazole-2-thiol (126 mg, 0.699 mmol), 4-chlorothieno[2,3-*d*]pyrimidine (102 mg, 0.601 mmol) and triethylamine (97 µL, 0.70 mmol) in DMSO (2 mL) was stirred for 60 min at 140 °C under microwave irradiation (200 W). Purification by column chromatography (dichloromethane/ethyl acetate 1:1) yielded a colorless solid (21 mg, 0.067 mmol, 11%).

Mp.: 151–153 °C; IR (KBr): $\tilde{\nu}$ [cm^{-1}] = 3430 (w, br, N-H), 3087 and 3050 (=C-H), 2949 (-C-H); ^{1}H-NMR (600 MHz, DMSO-d_6) δ [ppm] =3.81 (s, 3H, CH_3), 3.82 (s, 3H, CH_3), 6.89 (dd, J = 8.8, 2.5 Hz, 1H, arom. H), 6.95 (dd, J = 8.9, 2.4 Hz, 1H, arom. H), 7.01 (d, J = 2.5 Hz, 1H, arom. H), 7.22 (d, J = 2.4 Hz, 1H, arom. H), 7.41–7.49 (m, 3H, arom. H), 7.59 (d, J = 8.8 Hz, 1H, arom. H), 8.07 (dd, J = 6.0, 0.4 Hz, 2H, arom. H), 8.81 (s, 1H, arom. H), 8.82 (s, 1H, arom. H), 13.08 (s, 1H, NH), 13.14 (s, 1H, NH); ^{13}C-NMR: (151 MHz, DMSO-d_6) δ [ppm] = 55.46, 55.51 (CH_3), 94.04, 101.08, 112.02, 112.26, 113.89, 118.75, 118.77, 119.84, 129.58, 129.61, 152.56 (2C) (CH), 127.14, 127.25, 130.22, 136.44, 137.53, 138.28, 139.16, 144.52, 155.56, 156.80, 161.29, 161.53, 166.62, 166.66 (C); the spectra show two tautomeric forms of **8j** (ratio

approx. 2:1); MS (EI): m/z (%): 314.0 $[M]^{+\cdot}$ (100); HRMS (EI): calc. 314.02905, found 314.02897; HPLC (isocratic): 99.5% at 254 nm and 99.7% at 280 nm, t_{M+S} = 4.34 min, t_M(DMSO) = 1.06 min (ACN/water = 40:60), λ_{max} [nm] = 247, 298, 308; HPLC (gradient): 97.9% at 254 nm, t_{M+S} = 10.50 min, t_M(DMSO) = 1.26 min.

4-(3*H*-Imidazo[4,5-*b*]pyridine-2-ylthio)thieno[2,3-*d*]pyrimidine (**8k**)

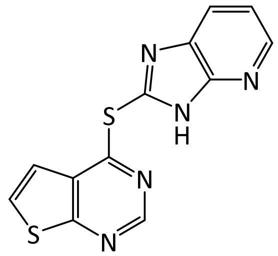

According to GP2, a solution of 4-chlorothieno[2,3-*d*]pyrimidine (102 mg, 0.601 mmol), 1*H*-imidazo[4,5-*b*]pyridine-2(3*H*)-thione[5] (91 mg, 0.60 mmol) and triethylamine (0.10 mL, 0.72 mmol) in DMF (2 mL) was stirred for 24 h at 120 °C. Instead of ice, water (20 mL) was used for quenching the reaction. After crystallization at 4 °C over night the isolated solid was further purified by column chromatography (toluene/ethanol 10:1). The product was obtained as a beige solid (18 mg, 0.063 mmol, 11%).

Mp.: 183-184 °C; IR (KBr): $\tilde{\nu}$ [cm^{-1}] = 3205 (w, br, N-H), 1512 (s); ^{1}H-NMR: (600 MHz, DMSO-d_6): δ [ppm] = 7.30-7.39 (m, 2H, arom. H, CH(pyridine)), 7.51-7.62 (m, 2H, CH (thiophene)), 7.92-8.20 (m, 4H, arom. H, CH(pyridine), CH(thiophene)), 8.34-8.56 (m, 2H, CH (pyridine)), 8.78-8.90 (m, 2H, CH (pyrimidine)), 13.50, 13.80 (2s, 2H, NH); ^{13}C-NMR: (151 MHz, DMSO-d_6) δ [ppm] = 118.5, 118.9 (2C), 119.0, 119.8, 127.0, 127.6, 127.7, 127.7, 127.9, 129.8, 129.9, 135.6, 142.5, 143.6, 144.6, 145.0, 149.2, 152.6, 155.6, 160.1, 160.5, 166.7, 166.8, the spectra show two tautomeric forms of **8k** (ratio approx. 2:1); MS (EI): m/z (%) = 285.0 $[M]^{+\cdot}$ (100), 227.0 $[M^{+\cdot}$-58] (57), 135.0 $[M^{+\cdot}$-150] (21); HRMS (EI): calc. for $C_{12}H_7N_5S_2$ 285.01374, found 285.01339; HPLC (isocratic): 99.2% at 254 nm and 99.5% at 280 nm, t_{M+S} = 3.14 min, t_M(DMSO) = 1.06 min (ACN/buffer$_{pH\ 2.7}$ = 35:65), λ_{max} [nm] = 232, 302; HPLC (gradient): 97.5% at 254 nm, t_{M+S} = 9.34 min, t_M(DMSO) = 1.28 min.

Details of thermodynamic solubility measurement

The thermodynamic aqueous solubility of selected compounds was determined using the established *shake flask*-method with subsequent readout via HPLC-UV.[6,7]

Calibration

Two stock solutions of each compound with a concentration of 10 mM (except for **8g**: 1 mM) were prepared. Three dilutions with ACN were prepared (concentration range: **3d**: 100 µM – 1 µM; **8c**: 2 mM – 100 µM; **8d**: 100 µM – 10 µM; **8e**: 1.8 mM – 100 µM; **8f**: 100 µM – 2 µM; **8g**: 10 µM – 0.3 µM; **8k**: 100 µM – 10 µM) from the stock solutions of the test compounds and measured as duplicates by HPLC (isocratic elution: solvent indicated in **Additional file 2, Table**, injection volume: indicated in **Additional file 2, Table**, flow rate: 1.000 mL/min, column temperature: 40 °C, time of detection: 8 min, detection by DAD at specific λ_{max} as indicated in **Additional file 2, Table**). The area under the curve (% AUC) of the substance peak was determined and together with the respective concentration a linear calibration graph was created with Microsoft Excel (2010, Microsoft Corporation) for every compound.

Additional file 2, Table Af1: Solvents for isocratic elution, injection volumes and specific λ_{max} for each compound

ID	solvent	injection volume [µL]	λ_{max} [nm]
**3d**	ACN/water (55:45)	10	293
**8c**	ACN/buffer$_{pH\ 2.7}$ (25:75)	7	240
**8d**	ACN/water (50:50)	10	231
**8e**	ACN/water (45:55)	10	231
**8f**	ACN/water (35:65)	10	231
**8g**	ACN/buffer$_{pH\ 2.7}$ (45:55)	20	289
**8k**	ACN/water (40:60)	10	303

Sample preparation and measurement of compound solubility

Samples of test compounds (0.25-1.0 mg) were placed in Whatman-Uni-Prep® vials and aqueous phosphate buffer, pH 7.4 (300 µL, composition is indicated in **Additional file 2, Table Af1**) was added. The presence of undissolved compound was a requirement to determine the thermodynamic solubility. The vials were capped with the appropriate filter, so that the filter did not touch the buffer solution. Additionally the vials were sealed with Parafilm M (Bemis Flexible Packaging, Neenah, WI, USA). The samples were incubated in an incubation shaker (IKA® KS 3000 ic control, Staufen, Germany) at 400 rpm and 25 °C for

24-72 h. The presence of undissolved compound was checked at 24 and 48 h. Measurement of the samples was performed after 24 h, 48 h and 72 h; he filter plunger was punched into the vial and the concentration in the supernatant was determined by HPLC using the same methods as for the calibration solutions (**Additional file 2, Table**). All samples were measured in triplicate. By determination of the area under the curve (% AUC) of the substance and the specific linear calibration graph, the concentration of every compound in aqueous buffer solution was calculated. By comparison of the samples after 24 h and 48 h it was checked if equilibrium had been achieved after 24 h; if not, the samples were compared after 48 h and 72 h to check if equilibrium had been achieved after 48 h. In the case of **3a** the concentration in the supernatant was below the limit of quantification. Therefore the lowest concentration (1 µM) used for the calibration was indicated as upper limit of solubility.

Additional file 2, Table Af1: Composition of phosphate buffer pH 7.4 for determination of aqueous solubility. Modified according to Ph. Eur.[8]

$Na_2HPO_4 \cdot 2\ H_2O$	290 mg
KH_2PO_4	20 mg
NaCl	808 mg
H_2O (bidest.)	*ad* 100 mL
12% aqueous HCl	*qs*[a]

[a] Adjustment to pH 7.4 was carried out using a pH meter equipped with a glass electrode.

References

1 Jansen, M.; Rabe, H.; Strehle, A.; Dieler, S.; Debus, F.; Dannhardt, G.; Akabas, M. H.; Lüddens, H., Synthesis of GABA A receptor agonists and evaluation of their α-subunit selectivity and orientation in the GABA binding site. *J. Med. Chem.* **2008**, *51*, 4430–4448.

2 Pissiotas, G.; Rohr, O.; Kristinsson, H., Heterocyclyloxyphenylureas, *Ger. Offen.* **1982**, DE 3145422 A1 19820701.

3 Ishige, O.; Nakamura, T.; Sato, N.; Kaneko, Y., Silver halide light-sensitive color photographic material, *Eur. Pat. Appl.* **1995**, EP 686871 A1 19951213.

4 Schlütke, L. M., Neue Purinderivate als Inhibitoren der Proteinkinase ALK. *Dissertation* Technische Universität Braunschweig **2016,** 169.

5 Chang, L.; Lee, S.-Y.; Leonczak, P.; Rozenski, J.; Jonghe, S. de; Hanck, T.; Müller, C. E.; Herdewijn, P., Imidazopyridine- and purine-thioacetamide derivatives: potent inhibitors of nucleotide pyrophosphatase/phosphodiesterase 1 (NPP1). *J. Med. Chem.* **2014**, *57*, 10080–10100.

6 Wölfel, S., *Neue Inhibitoren der Anaplastic Lymphoma Kinase*, **2014**. Shaker Verlag, Aachen, 77-80.

7 Glomme, A.; Marz, J.; Dressman, J. B., Comparison of a miniaturized shake-flask solubility method with automated potentiometric acid/base titrations and calculated solubilities. *J. Pharm. Sci.* **2005**, *94*, 1–16.

8 *Europäisches Arzneibuch.* 8. Ausgabe, einschließlich 1. bis 6. Nachtrag, **2014**. Deutscher Apotheker Verlag, Stuttgart, 6768.
