# Supplementary figures and images for: Antiplasmodial dihetarylthioethers target the coenzyme A synthesis pathway in Plasmodium falciparum erythrocytic stages
Source: Malar J. 2017 May 15;16:192. doi: 10.1186/s12936-017-1839-3 (PMC5430599; doi:10.1186/s12936-017-1839-3)

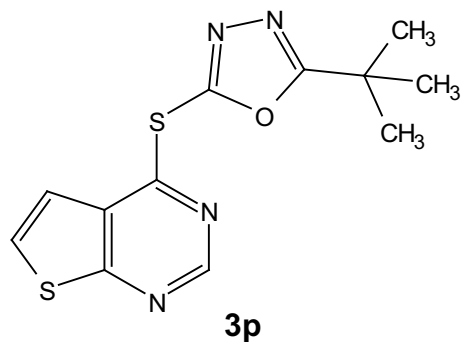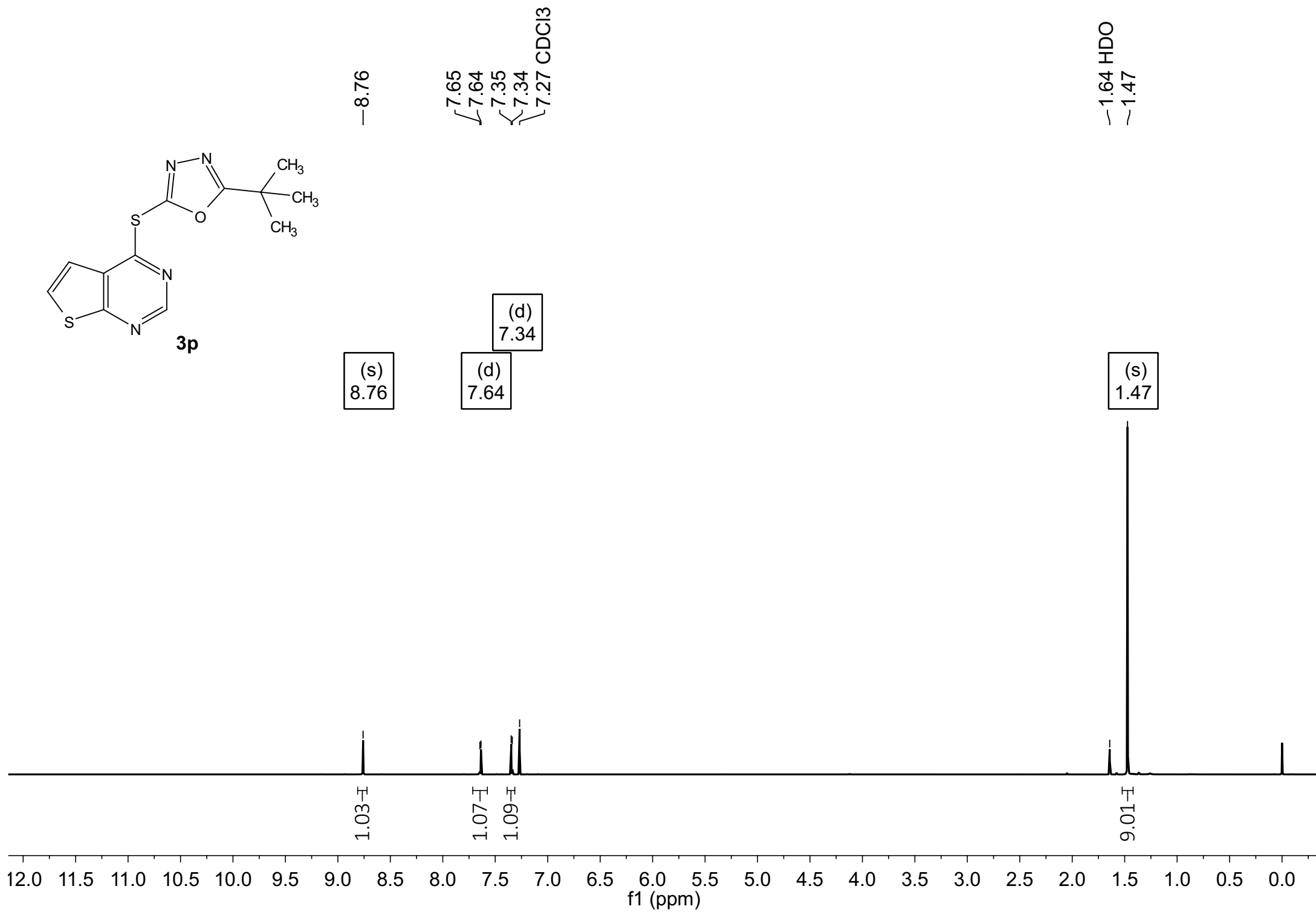

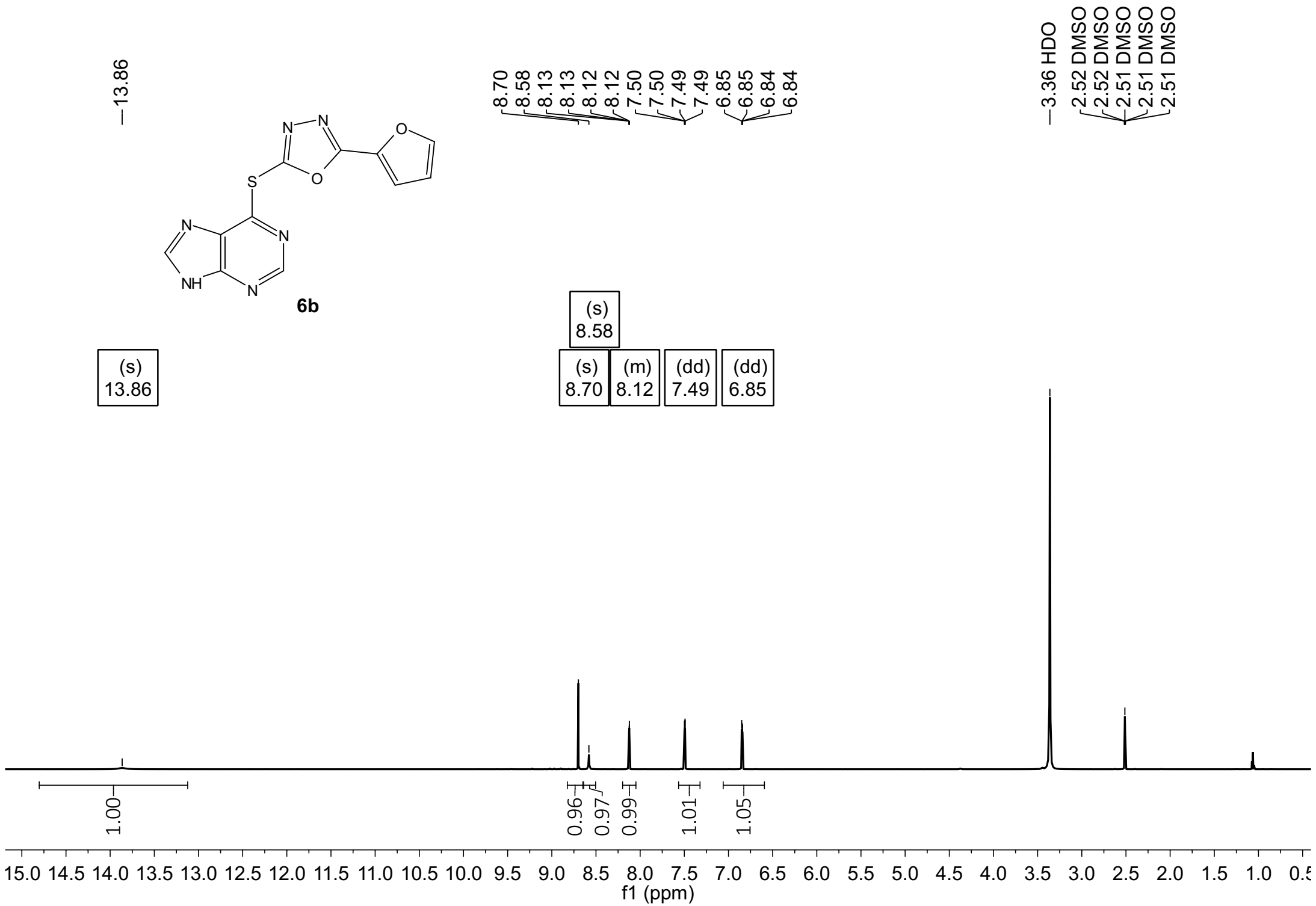

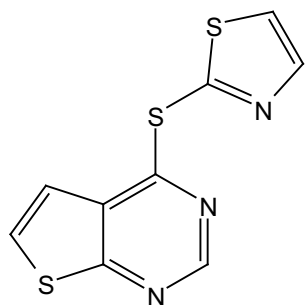

**8d**

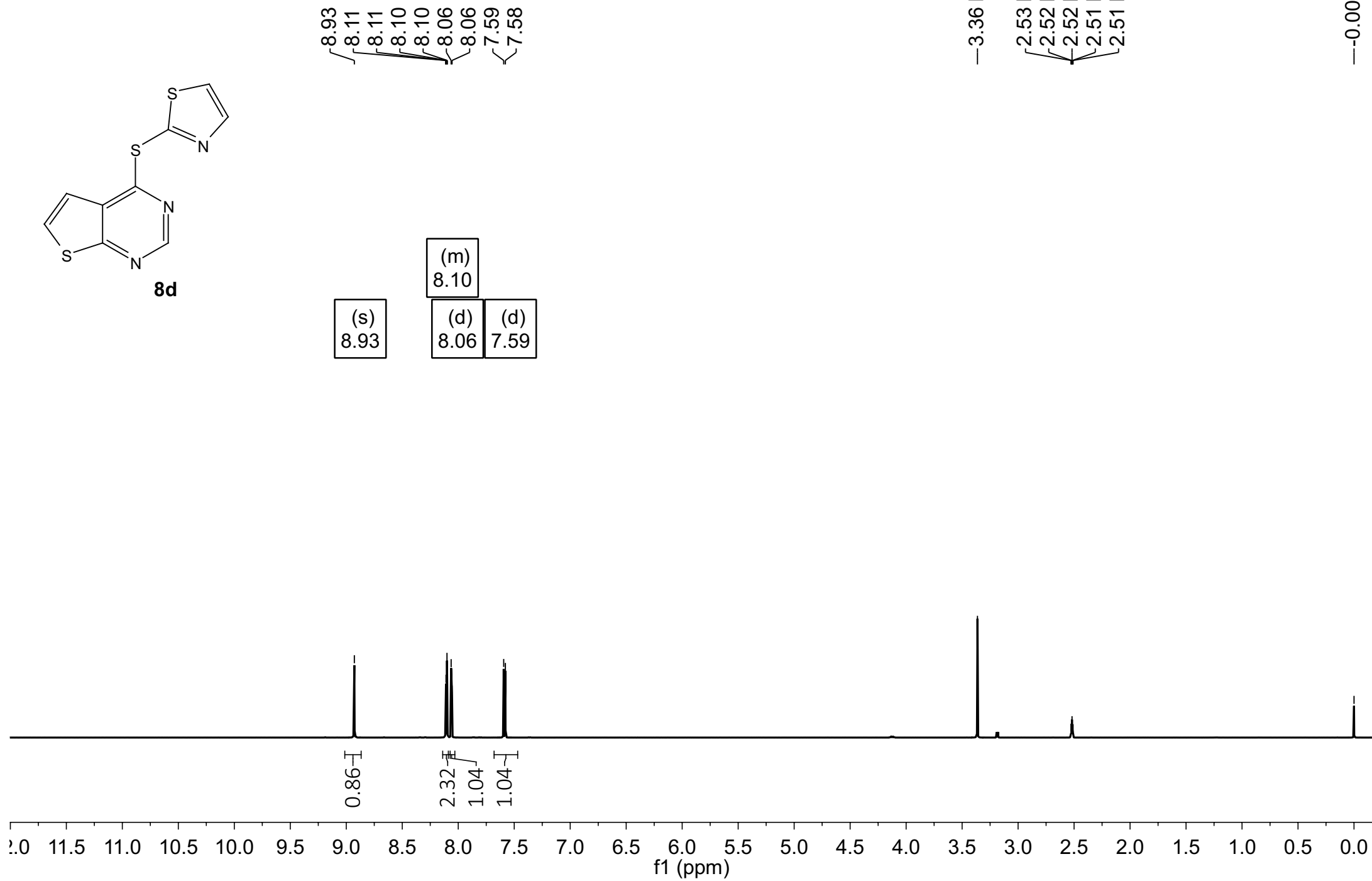

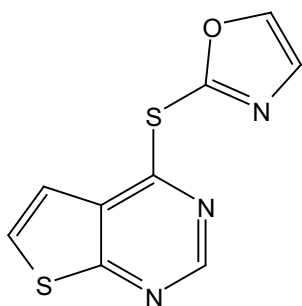

**8e**

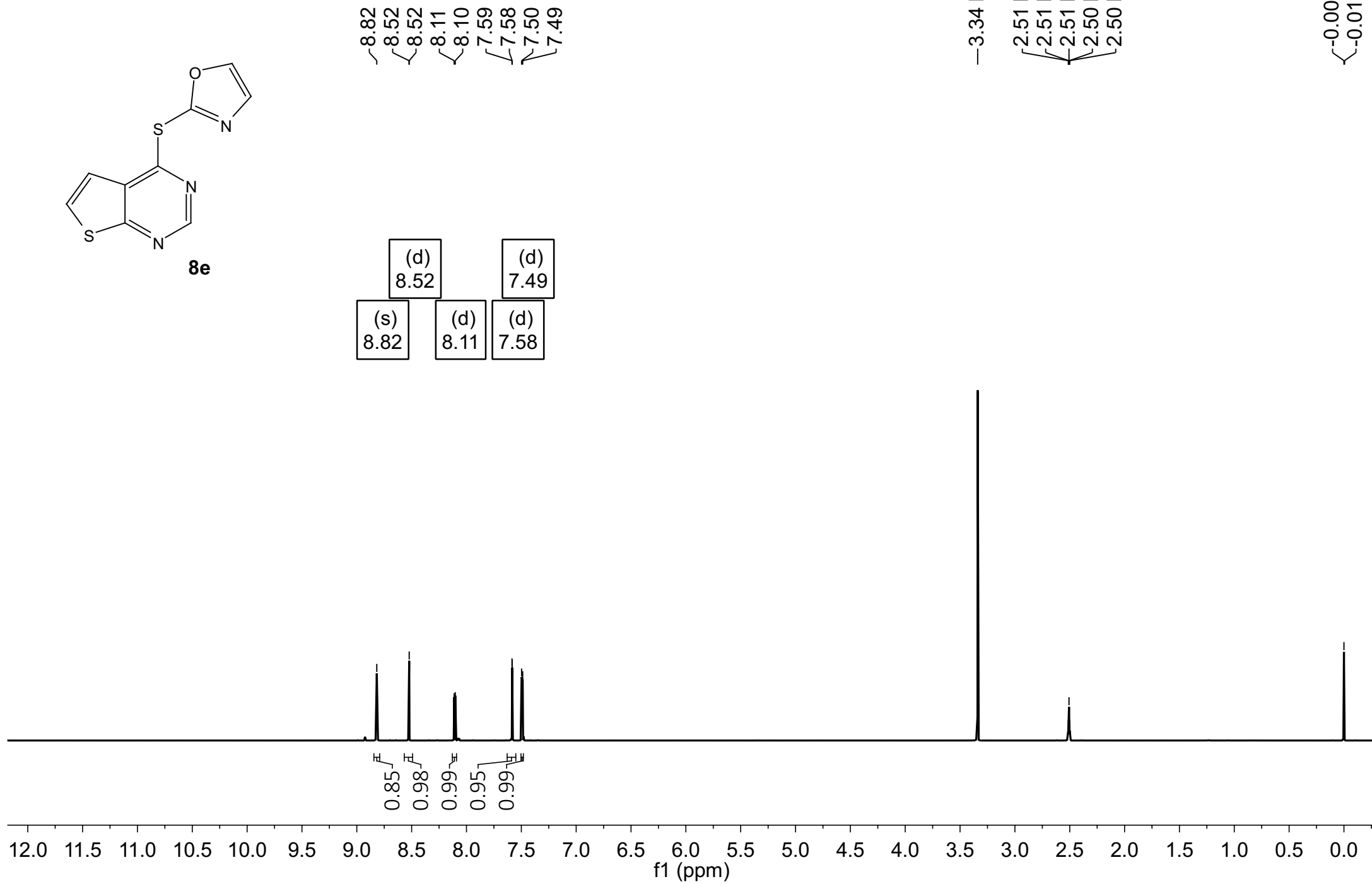

Supplement: Supplementary file 3 — Additional file 3. 1H NMR spectra of test compounds 3p, 6d, 8d, 8e. [file 12936_2017_1839_MOESM3_ESM.pdf]
