# Supplementary material for: Antiplasmodial dihetarylthioethers target the coenzyme A synthesis pathway in Plasmodium falciparum erythrocytic stages
Source: Malar J. 2017 May 15;16:192. doi: 10.1186/s12936-017-1839-3 (PMC5430599; doi:10.1186/s12936-017-1839-3)
Supplement: Supplementary file 4 — Additional file 4. Dose–response plots for test compounds and reference drugs showing parasite viability (3D7) in absence and presence of coenzyme A. [file 12936_2017_1839_MOESM4_ESM.pdf]

Thomas Weidner, Leonardo Lucantoni, Abed Nasereddin, Lutz Preu, Peter G Jones, Ron Dzikowski, Vicky M Avery and Conrad Kunick: Antiplasmodial dihetarylthioethers target the coenzyme A synthesis pathway in *Plasmodium falciparum* erythrocytic stages

Additional File 4

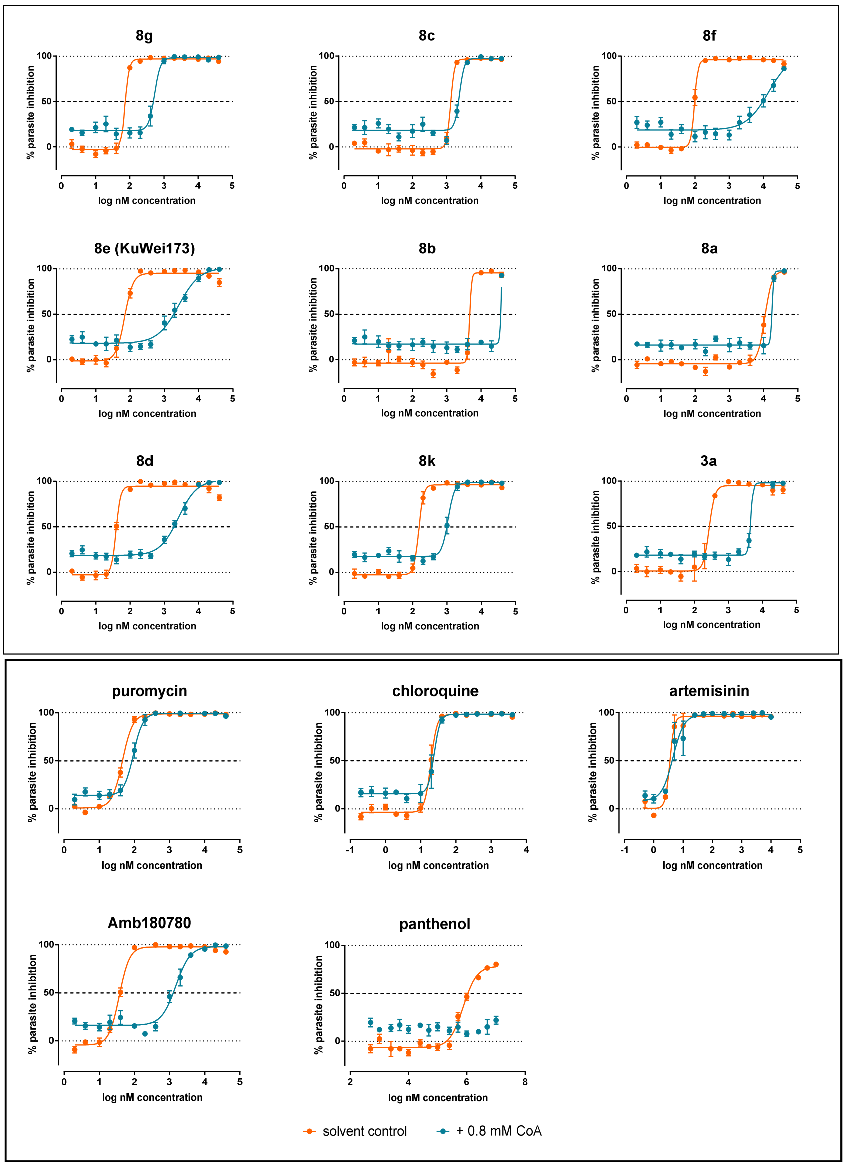

Figure Af4-1: Dose-response plots for test compounds (top) and reference drugs (bottom) showing parasite viability (3D7) in absence and presence of coenzyme A.

Table Af4-1: Assay performance: all Z’ >>0.5 [1]

	Z' point 1	Z' point 2	mean Z'	SEM Z'
solvent A	0.75	0.82	0.78	0.03
solvent B	0.84	0.74	0.79	0.05
rescue A	0.76	0.78	0.77	0.01
rescue B	0.77	0.78	0.77	0.00

1. Zhang JH, Chung TD, Oldenburg KR: **A Simple Statistical Parameter for Use in Evaluation and Validation of High Throughput Screening Assays.** *Journal of biomolecular screening* 1999, **4:**67-73.
